# Advancements in real-time oncology diagnosis: harnessing AI and image fusion techniques

**DOI:** 10.3389/fonc.2025.1468753

**Published:** 2025-11-26

**Authors:** Leila Bagheriye, Johan Kwisthout

**Affiliations:** Donders Institute for Brain, Cognition and Behaviour, Radboud University, Nijmegen, Netherlands

**Keywords:** real-time, image fusion, cancer, diagnosis, artificial intelligence, spectroscopy, hardware accelerator, hyperspectral imaging

## Abstract

Real-time computer-aided diagnosis using artificial intelligence (AI), with images, can help oncologists diagnose cancer with high accuracy and in an early phase. It explores various real-time techniques, encompassing technical solutions, AI-based imaging, and image fusion diagnosis. Techniques such as computer-aided surgical navigation systems and augmented reality platforms have improved the precision of minimally invasive procedures once combined with real-time imaging and robotic assistance. The integration of modalities like ultrasound into image fusion workflows improves procedural guidance, reduces radiation exposure, and provides high cross-modality interpretation. Optical imaging techniques—such as diffuse reflectance spectroscopy, Raman spectroscopy, fluorescence endoscopy, and hyperspectral imaging—are emerging as powerful diagnostic tools for tumor detection, margin assessment, and intraoperative decision-making. Promising methods like fluorescence confocal microscopy and shear wave elastography offer practical, real-time diagnostic capabilities. However, regarding these technologies, there are technical challenges including tissue motion, registration variability, and data imbalance. The incorporation of AI-based landmark detection and the development of robust algorithms will be key to overcoming these barriers. We close by offering a more futuristic overview to solve existing problems in real-time image-based cancer diagnosis. The reviewed technologies altogether mark that continued research, multi-center validation, and providing hardware accelerators will be crucial to their full clinical potential and usage.

## 1 Introduction

REAL-TIME medical image-based cancer diagnosis utilizing artificial intelligence (AI) has recently gained widespread use in oncology. It has been shown that real-time imaging improves the diagnosis, treatment, and follow-up process of patients. Studies have demonstrated that to achieve a satisfactory patient-specific diagnostic and therapeutic process, combining pretreatment clinical variables with computer-aided intraoperative imaging information is essential (1). Image fusion shows considerable potential in aiding tumor diagnosis, directing biopsies, and facilitating interventional ablations, adding accuracy in the operating room (2). The process of image fusion, which involves directly comparing the target lesion with other images, helps multidisciplinary teams achieve clearer visualization for surgical or radiation treatment planning. A fusion system combining transrectal ultrasound (TRUS) with magnetic resonance imaging (MRI) could address the limitations of relying solely on 2D grayscale TRUS for intraoperative guidance (1). The fusion of endoscopic ultrasound (EUS) and computed tomography (CT) images allows for easier navigation and profiling of target tumors and adjacent anatomical structures (2). This integration method can significantly shorten the learning process for mastering and navigating EUS procedures. MRI-ultrasound (US) image fusion has been utilized for prostate biopsy using the ARTEMIS semi-robotic fusion biopsy system (3). MR-US technology, as a multiparametric approach along with semi-robotic, electronic–mechanical tracking of the prostate, helps clinicians precisely navigate needles to identify lesions, subsequently improving the accuracy of the biopsy. The system utilizes motion compensation to align previously constructed virtual images with real-time US images as the physician navigates the needle to a target using the probe and mechanical arm. This method enhances the ability to target regions of interest (ROIs) and, in systematic biopsies, helps prevent both under-sampling and over-sampling.

Optical imaging and spectroscopic techniques, while having limited penetration, are well suited for examining surface epithelial tissues for early cancer detection. There are optical techniques such as diffuse reflectance spectroscopy (DRS), autofluorescence spectroscopy (AFS), and autofluorescence imaging which provide non-destructive and exogenous agent-free tissue sensing (4). Integrating DRS technology into the surgical workflow for tumor mapping can be facilitated through augmented reality or an optional heatmap overlay added to a laparoscopic or robotic camera feed, which only minimally deviates from the standard plan of care. A concurrent *in vivo* validation of the real-time tracking technology and classification is planned, requiring adaptation of the probe tracking system. Advanced deep learning neural networks can assist in accurate probe tip location detection and tracking in DRS. Correlating real-time tissue classification in DRS with clinical outcomes such as recurrence, positive resection margin rate, or overall survival will be necessary to demonstrate the clinical utility of this technology before its potential clinical adoption (5, 6).

Utilizing hardware acceleration platforms such as GPUs or FPGAs to implement the full brain cancer detection algorithm is possible (7). This implementation should explore the design space to achieve the optimal balance between real-time execution, memory usage, and power dissipation using heterogeneous platforms. On the other hand, various AI techniques have been employed for cancer diagnosis within real-time imaging/image fusion, but the lack of external validation poses a significant obstacle to the secure and regular utilization of AI classification models in clinical practice. Therefore, to take an essential step toward the clinical application of this technology, the classification model needs external validation on a separate cohort of patients.

In this review, we specifically examine modalities that have been applied in real-time AI-based cancer diagnosis. For each technique, we detail not only its clinical application but also its technical parameters and maturity level. **Table 1** shows the paper structure as follows. Section II discusses real-time US-based imaging and image fusion. Section III introduces real-time *in vivo* cancer diagnosis with different spectroscopy techniques and real-time optical imaging-based cancer diagnosis methods. Section IV explains elastography-based cancer diagnosis real time methods. Section V describes different real-time fluorescence image-based cancer diagnosis techniques. Real-time hyperspectral imaging-based cancer diagnosis is covered in Section VI. Section VII describes PET/MRI image deep learning for real-time breast cancer. In section VIII, we provide an overall discussion of the different approaches and future work. Finally, Section IX concludes the paper.

**Table 1:** Real-time imaging modalities.

Section	Subsection
II. Real-time ultrasound-based image fusion for oncology	A. MRI–US fusion for prostate biopsy B. Endoscopic/transabdominal fusion techniques C. Liver ultrasound fusion approaches
III. Real-time spectroscopy and optical imaging techniques	A. Raman spectroscopy in GI and lung cancer B. Mass spectrometry pens C. Diffuse reflectance spectroscopy D. Optical coherence tomography (OCT) E. Polyp detection and classification with optical tools
IV. Real-time elastography and mechanical imaging	A. Shear-wave elastography B. Video-based invasion depth assessment
V. Neuromorphic AI systems for cervical cancer diagnosis	
VI. Real-time fluorescence imaging and optical navigation	A. Augmented reality systems (GAINS) B. Fluorescence lifetime endoscopy C. Digital biopsy tools
VII. Real-time hyperspectral-imaging-based cancer diagnosis	A. VNIR–NIR HSI fusion B. Skin cancer with mm-wave imaging C. Brain tumor delineation D. Esophageal cancer diagnosis
VIII. Radiomics and deep learning in multimodal imaging	A. PET/MRI fusion for breast cancer B. AI-based radiomics in CT/MRI C. AI-based image-omics integration
IX. Discussion	
X. Conclusion	

## 2 Real-time ultrasound image fusion for oncology

In this section, US-based imaging will be covered. Online US–MRI-based image fusion, real-time transabdominal and endoscopic US, and US imaging of the liver will be described.

### 2.1 Real-time MRI-US image fusion for prostate biopsy

**Figure 1:**
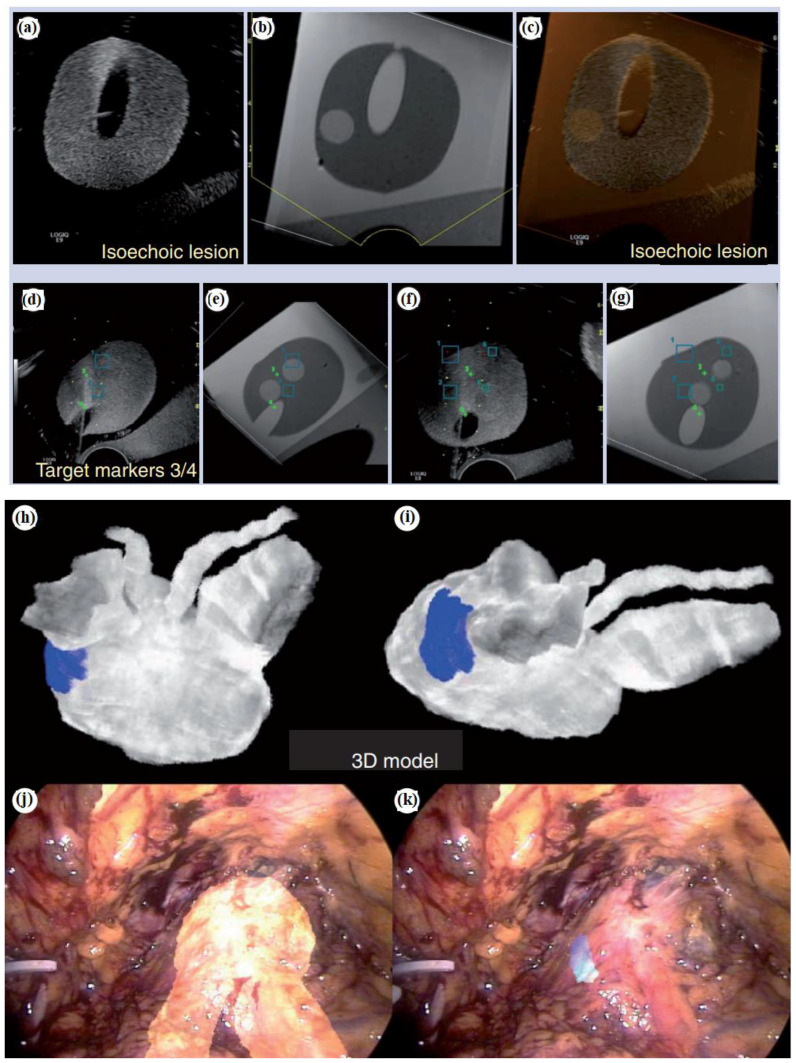
Fusion platform for hybridizing transrectal ultrasonography (TRUS) with magnetic resonance imaging (MRI). **(a)** For axial TRUS image in real-time mode, there is no detectable lesion. **(b)** The lesion on the right side is visualized with the synchronized, previously acquired MR image. **(c)** The MR overlays onto the TRUS as image fusion to facilitate the determination of the lesion which is visible only on the MR. **(d)** Real-time TRUS visualization of the hyperechoic needle (with a GPS-like needle tracking system) documents with spatial digital data the precise location of each biopsy sample. **(e)** Digital marks are used to register the spatial coordinates of both the distal and proximal endpoints of the biopsy trajectory. **(f)** Subsequently, for spatial re-targeting, real-time TRUS guidance is employed. **(g)** With GPS-like computer-based marking of the previous biopsy position, biopsy location is determined using newly acquired 3D volume data (1). Augmented reality navigation in laparoscopic radical prostatectomy: **(h)** and **(i)** show views from different angles. In order to determine the blue-colored cancer area (which is proved with biopsy) on the left-posterior-lateral surface of the prostate, intraoperatively, the workstation reconstructed a 3D prostate surgical model. **(j)** The 3D model of the prostate and seminal vesicles are overlaid onto the real endoscopic view with augmented reality techniques. **(k)** The cancer lesion is overlaid on the left-posterior-lateral prostate (the 3D model) (1). Reproduced from (1), with permission from Taylor & Francis.

To enhance the precision of prostate biopsy, various approaches can be considered. These include merging US images with MRI, integrating contrast-enhanced US, utilizing elastography, combining 3D US with computer-assisted techniques, and utilizing robotic based needle guidance (1). TRUS imaging has primarily served as a basic guidance tool for systematic biopsy. Its role is to ensure that biopsies are taken from all sextant areas of the prostate in a random manner. Additionally, a small number of biopsies are taken by targeting suspicious lesions directly. The fusion of real-time TRUS with preoperative MRI has been introduced (**Figure 1**). The goal is to compensate for the limitations of current 2D grayscale TRUS alone for intraoperative guidance to enhance the precision of targeted prostate biopsy and prostate intervention (1). In **Figure 1a**, no detectable lesion is shown in the real-time axial TRUS image, while **Figure 1b** on the right side shows the lesion with the synchronized, previously acquired MR image. In **Figure 1c**, the MR is overlaid onto the TRUS (image fusion), aiding in the targeting of the lesion visible only on the MRI. **Figure 1d** shows real-time TRUS visualization of the hyperechoic needle using a needle tracking system like a global positioning system (GPS), allowing the precise documentation of the exact location of each biopsy sample with spatial digital data. In **Figure 1e**, digital marks were used to register the spatial points of both the distal and proximal endpoints of the biopsy trajectory in the 3D MR volume data of the phantom. **Figure 1f** shows fusion with GPS-like technology. In **Figure 1g**, later on, real-time TRUS guidance is utilized for spatial re-targeting. **Figure 1f** illustrates the ability to revisit a previously marked biopsy location using newly acquired 3D volume data. This is achieved through computer-based marking, like GPS, of the previous biopsy position. On MRI of 52 suspected cases of prostate cancer, the TRUS/MRI hybrid system detected cancer in 26 patients and improved identification in 14 of these 26, a 61% higher positive rate than systematic biopsy alone (8). The system requires no major workflow changes and can be used outside the MRI suite with only an added workstation, integrating preoperative MRI-DICOM data with the TRUS probe and US machine.

Moreover, **Figures 1h–k** show augmented reality navigation in laparoscopic radical prostatectomy. Augmented reality integrates intraoperative surgical navigation to overlay a 3D image onto the live surgical view. This technology displays 3D anatomy beyond the surgical view, revealing the anatomical orientation of the targeted pathology and surrounding tissues before surgical exposure (1). Augmented reality involves technical steps including image acquisition, segmentation, registration, visualization, and navigation. These can be integrated with energy-based ablative machines and robotics. **Figures 1h,i** show views from different angles. The workstation created a 3D surgical model of the prostate from original intraoperatively acquired real-time 2D transrectal ultrasonography imagery, demonstrating the area of biopsy-proven cancer, indicated by a blue color (which is located on the left posterior-lateral surface of the prostate). As shown in **Figure 1j**, via augmented reality techniques, the 3D model of the prostate and seminal vesicles are overlaid onto the real endoscopic view. **Figure 1k** shows the 3D model of the cancerous lesion overlaid on the left posterior-lateral aspect of the prostate. In (9), for augmented reality overlay, a stereo-endoscopic visualization system has been described. Without the need for external tracking devices, it allows 3D-to-3D registration of the preoperative CT scan with the real surgical field. As a result, the integration of stereoscopic vision into robotic systems represents a significant advancement in augmenting surgeons’ human senses during image-guided surgery.

### 2.2 Real-time endoscopic/transabdominal fusion techniques

To enhance the diagnosis and interventional treatment of focal liver lesions, real-time US fusion for the liver is emerging as a promising approach over traditional US methods.

Image fusion provides considerable benefits for pinpointing liver lesions in minimally invasive procedures like biopsies, percutaneous ablations, or planning radiation treatments (2). Simpler navigation and characterization of the target tumor and nearby anatomical structures can be achieved by combining EUS and CT images.

**Figure 2:**
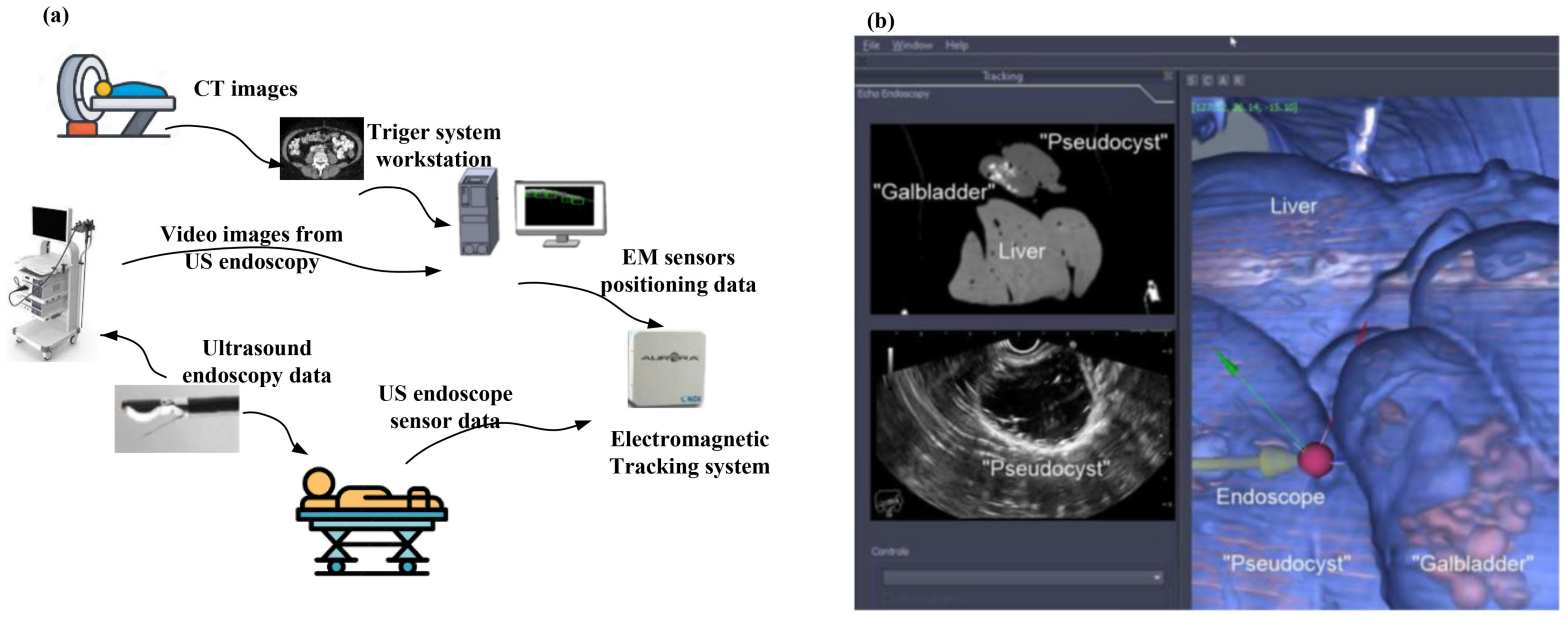
**(a)** A typical image fusion framework. A virtual 3D map of the patient is created based on pre-procedure CT images. Subsequently, to overlay the EM sensor locations onto the 3D model, the image fusion software is used. To identify the clinical target, live EUS images are merged with the 3D model (2). **(b)** Real-time image fusion (EUS/CT) through which the 3D reconstruction of the segmented CT has been done (featuring a phantom “gallbladder with stones,” “pseudocyst,” and liver). The oblique section represents the co-registered large-field CT, which is showing all three organs. The narrow EUS image shows the pseudocyst [adapted from (2)]. Reproduced from (2) ([Journal]), licensed under CC BY 4.0.

A typical image fusion session, as shown in **Figure 2a**, is composed of the electromagnetic (EM) field generator, external physical markers, and the image fusion software (2). The EM tracking and field generator system is positioned near the patient and connected to the computer intended for running the fusion imaging software. Then, active marker disks are placed on the patient’s xiphoid process.

The EM sensor is inserted into the navigation catheter within the working channel of the endoscope or echoendoscope (2).

To generate a 3D model of the patient’s anatomy, pre-procedure CT scans are imported into the fusion imaging (FI) software. To identify the target, dual visualization of the EUS image and its corresponding virtual section through the CT volume is employed.

After reaching the target, the EUS position is fixed. To collect a biopsy, the navigation catheter is withdrawn and substituted with a fine aspiration needle (2).

**Figures 2b–k** show the software platform for EUS/CT image fusion, which provide automatic processing of CT for 3D rendering and segmentation, nodule detection, organ/tumor segmentation, registration of patient’s CT for endoscopy procedures, localization and tracing of therapeutic devices using tracking technologies, virtual visualization of medical instruments on the CT stack, and augmented reality for virtual visualization of the patient’s anatomy over intraprocedural video (2). An earlier version of the software underwent testing using a specially constructed model of pig organs.

At first, a CT scan of the model was conducted using a specific pancreatic protocol. The images were then segmented, and 3D reconstruction was done before uploading into the software. This enabled the visualization of the echoendoscope’s position within the 3D CT cube and real-time movement of the same section.

The integration of real-time EUS with CT significantly improved the visualization of specific lesions or organs, enabling complex therapeutic procedures and improving operator confidence. **Figure 2b** (image fusion (EUS/CT) testing) shows the 3D reconstruction of the segmented CT scan, featuring a simulated gallbladder with stones, a pseudocyst, and the liver, illustrating real-time EUS-CT fusion. The oblique section displays the co-registered large-field CT, showing all three organs, alongside the narrow EUS image, which only shows the pseudocyst.

### 2.3 Real-time liver ultrasound fusion approaches

US imaging of the liver is frequently employed in clinical practice to diagnose and treat liver diseases, providing various liver images (10). However, inconsistencies exist between CT scans, MRIs, and liver US images when evaluating focal liver lesions, as CT or MRI scans provide cross-sectional views, including coronal and sagittal plane images. For an accurate identification of the areas of interest in these images, it is necessary to mentally align CT or MRI images with the planes corresponding to real-time B-mode ultrasound locations. While liver US does not require additional instruments, considerable training and experience are needed, as operator errors can occur.

Air in the lungs and intestinal gas present during liver ultrasound scans may obstruct the acoustic window, possibly masking small or inconspicuous foci in difficult-to-observe regions. The initial steps in real-time US fusion imaging of the liver entail preparing and positioning the patient along with deploying electromagnetic field equipment and a US machine.

To achieve high accuracy in comparisons during real-time US fusion imaging, it is necessary for the patient to maintain a relaxed posture to minimize movements. Image fusion begins with retrieving stored CT or MRI images. By initiating the electromagnetic field generator, the US machine acquires dynamic US images. The spatial information in the CT or MRI images and US images is adjusted based on the positioning point, plane, and 3D data to merge the two sets of images and display them in real time. The fusion US is then carried out.

The operator positions the US probe to capture images in a plane that corresponds to one of the previously acquired CT or MRI images, usually in a cross-sectional plane, and designates this plane as the matching coordinates. The matching process relies on 3D information, where a computer compares and aligns the captured data with the 3D-reconstructed spatial coordinates derived from CT or MRI images. Research indicates that image fusion improves the detection and assessment of focal liver lesions. Using real-time US combined with CT or MR images, the detection rate of small hepatocellular carcinomas (<3cm) rose from 78.8% to 90.5% (2).

## 3 Real-time spectroscopy and optical imaging techniques

In this section, different spectroscopy technologies will be explained. To this end, Raman spectroscopy is used in real-time cancer diagnosis, a handheld mass spectrometry system is used in cancer diagnosis, and diffuse reflectance spectroscopy and real-time endoscopic Raman spectroscopy are used in gastrointestinal cancers. Moreover, optical-based diagnosis, including real-time optical-based diagnosis of colorectal cancer, optical diagnosis of neoplastic polyps during colonoscopy, and using pattern recognition and optical coherence tomography (PR-OCT) for real-time diagnosis of colorectal cancer will be described.

### 3.1 Real-time Raman spectroscopy in GI and lung cancer

Histopathological analysis of tissue samples obtained through biopsy stands as the gold standard for diagnosing cancer (4).

The diagnostic procedure is lengthy, causes anxiety, and extends the overall duration of the process. It is very important to avoid unnecessary biopsies. However, patients cannot afford to overlook cancerous lesions that require biopsy. Accurately diagnosing cancer in real time and *in vivo*, with high sensitivity and specificity, while sparing most benign lesions poses a significant challenge. For this purpose, Raman spectroscopy has emerged as a practical tool for rapid *in vivo* tissue diagnosis. Subtle biochemical compositional changes are captured by Raman technique in the early stages of tissue malignancy before any visible morphological changes occur. Without taking a biopsy, by directing a laser beam directly onto suspected cancerous lesions, *in vivo* Raman spectra are measured. Important developments such as proprietary Raman spectrometer designs enhance spectral signal-to-noise ratios and decrease the necessary integration time to just a few seconds. The advancement of data mining and bioinformatics research significantly streamlines the automated analysis of Raman spectra.

**Figure 3:**
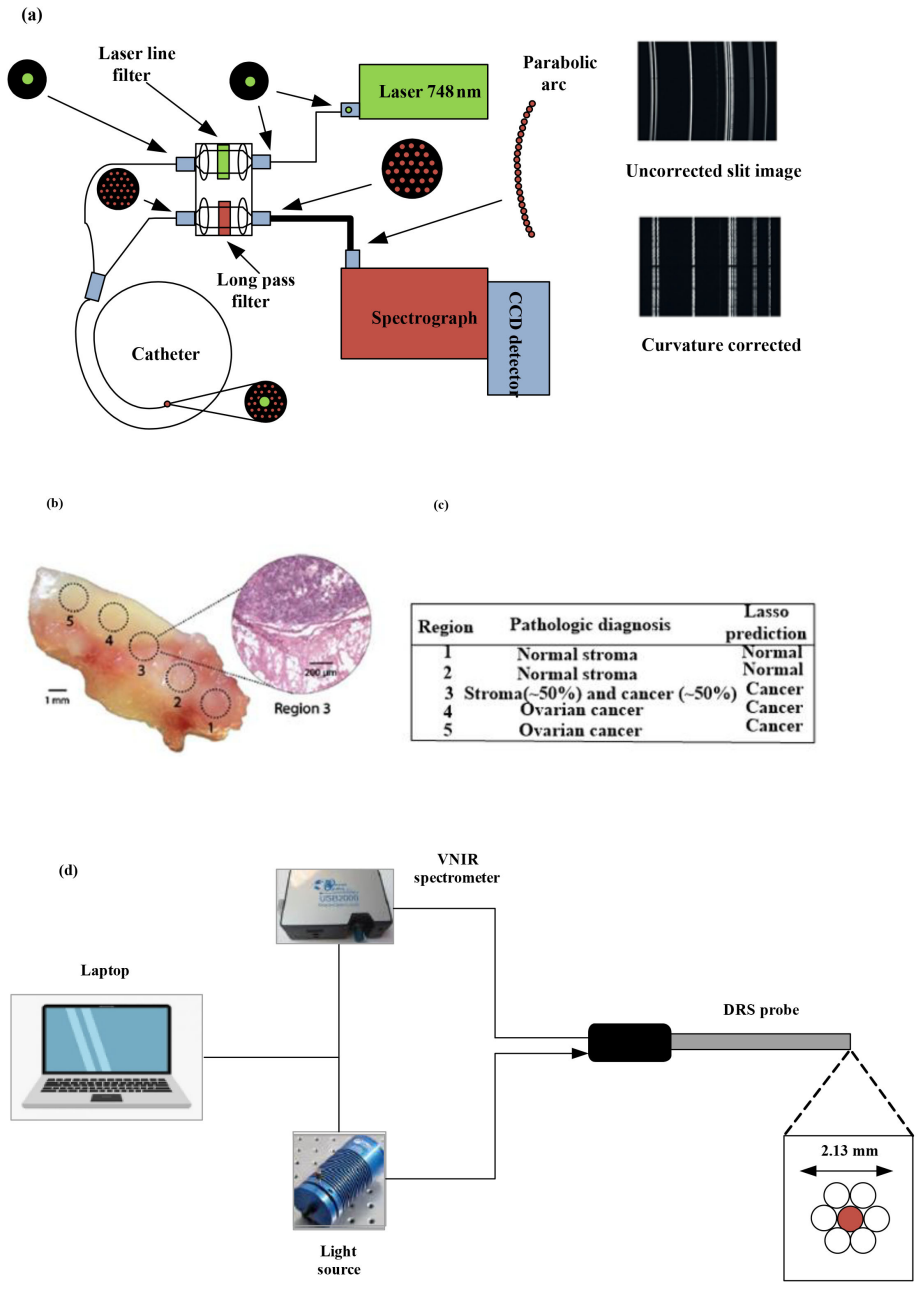
**(a)** Diagram illustrating the high-speed, real-time Raman system designed to diagnose skin cancer [(4)]. **(b)** A mixed histologic composition in a highgrade serous ovarian cancer (HGSC) tissue sample analyzed with the MasSpec Pen. MasSpec Pen-based analysis on optical image of the tissue sample at regions 1 to 5, followed by freezing, sectioning, and hematoxylin and eosin (H&E) staining. The inset shows the H&E-stained tissue section from region 3. It reveals a mixed histologic composition that includes both cancer and adjacent normal stroma tissue. **(c)** The diagnosis of the five regions is shown in the table alongside the Lasso prediction results [reproduced from (24) Science Translational Medicine (Sci Transl Med). with permission from American Association for the Advancement of Science.]. **(d)** Setup and instrumentation for diffuse reflectance spectroscopy (DRS) for data acquisition and sample illumination. The DRS probe was connected to both the light source and the spectrometer. All electronic devices communicated with the software (11).

Raman spectroscopy, through providing high molecular specificity, effectively reduces the occurrence of false-positive biopsies in cancer diagnosis. **Figure 3a** depicts a schematic of a real-time Raman spectrometer designed for skin cancer detection. Without correction, the image captured by the charge-coupled device (CCD) camera with a straight entrance slit exhibits curvature. Arranging the optic fiber array in a reverse orientation effectively corrects the curvature.

Different methods have been used in Raman analysis such as naïve Bayesian classifiers (NBC), linear discriminant analysis (LDA), support vector machine (SVM), and artificial neural network (ANN) (12). Training a Bayesian classifier is straightforward, and it performs well even when the assumption of feature independence is not met (13).

Besides assuming conditional probability density functions, decision hyperplanes can be computed to partition the feature space into regions for each class. With Raman spectroscopy, LDA is the most popular technique for classifying malignant tissues (14, 15). Generalized discriminant analysis (GDA) maps the original variables into a new feature space where the variables are nonlinearly correlated with the originals (16). Both LDA and GDA are frequently employed together with principal component analysis (PCA) or partial least squares (PLS) (17, 18). For the classification of cancerous tissues using Raman spectroscopy, SVMs have been reported (19, 20). For SVMs, optimal generalization performance is attained with high-dimensional data and/or datasets with a low ratio of training samples to input dimensionality (21). When data follows a normal distribution, LDA typically offers good generalization compared with SVMs, which require tuning numerous parameters and may lead to overfitting (21). Training neural network classifiers demands intensive computation and encounters issues such as converging to local minimum and susceptibility to outliers (22). For the classification of Raman spectra for tissue characterization, neural networks are used rarely (23).

### 3.2 Real-time cancer diagnosis with mass spectrometry pens

Traditional approaches to histopathological tissue diagnosis require significant labor and time, often causing delays in decision-making for diagnostic and therapeutic interventions. A recently reported advancement involves the creation of MasSpec Pen, a handheld mass spectrometry device designed for swift and noninvasive diagnosis of human cancer tissues, utilizing automation and biocompatible materials (24). For efficient extraction of biomolecules, the MasSpec Pen enables precise and automated dispensing of a defined water droplet onto tissue surfaces. The MasSpec Pen was employed for *ex vivo* molecular analysis. It examines 20 thin tissue sections from human cancer specimens and 253 tissue samples from patients (24). These samples comprised both normal and cancerous tissues from a range of organs, including the breast, lung, thyroid, and ovary.

The mass spectra acquired displayed rich molecular profiles containing a range of potential cancer biomarkers, such as metabolites, lipids, and proteins (24). Statistical classifiers, based on molecular data validated through histological methods, enabled accurate cancer predictions with high sensitivity, specificity, and overall accuracy. These classifiers were also capable of distinguishing between benign and malignant thyroid tumors as well as identifying different histologic subtypes of lung cancer. The classifier-enabled cancer diagnosis with high accuracy even in marginal tumor regions displaying mixed histologic composition.

It has been shown that the MasSpec Pen is applicable for *in vivo* cancer diagnosis during surgery in tumor-bearing mouse models, accomplishing this without inducing any discernible tissue damage or stress to the animal. MasSpec Pen has shown its high potential to be employed as a clinical and intraoperative technology in *ex vivo* and *in vivo* cancer diagnosis (24).

To build molecular databases, averages of three mass spectra were used. The full mass range of the spectra was partitioned, and four representative mass spectra were analyzed for PCA for each tissue section. For tissue classification, the least absolute shrinkage and selection (LASSO) method was applied. Since LASSO yields “sparse” models, the generated models are easier to interpret compared with other regularization methods. The LASSO method computes mathematical weight for each statistically significant feature, determined by the feature’s importance in characterizing a specific class in mass spectral analysis.

To pinpoint histologically unique areas within a solitary human tissue sample featuring high-grade serous ovarian cancer (HGSC) alongside normal ovarian stroma tissue, we assessed the efficacy of the MasSpec Pen. The MasSpec Pen sequentially analyzed five regions of the tissue sample, as determined in the optical image shown in **Figure 3b**. The regions were labeled from 1 to 5.

Following the MasSpec Pen analysis, the tissue sample underwent freezing, sectioning, and staining with hematoxylin and eosin (H&E). The inset displays an optical image of the H&E-stained tissue section from region 3, revealing a mixed histologic composition that includes both cancerous and adjacent normal tissue (24). **Figure 3c** describes the pathological diagnosis of the five regions alongside the LASSO prediction outcomes.

### 3.3 Real-time cancer diagnosis with diffuse reflectance spectroscopy

Upper gastrointestinal tract cancers continue to be a major factor in the worldwide cancer burden. Precisely identifying tumor margins is essential for effective cancer removal and enhancing overall survival rates. Current real-time mapping techniques do not allow for a full resection margin assessment. Therefore, it is essential to evaluate the capacity of diffuse reflectance spectroscopy (DRS) to distinguish between tissue types and offer real-time feedback to operators on gastric and esophageal cancer specimens. Research has explored the creation of a DRS probe and tracking system, showing that machine learning can accurately differentiate between normal and cancerous tissue in real time for gastric and esophageal samples (11). **Figure 3d** illustrates the DRS instrumentation used for *ex vivo* data collection. The DRS probe was linked to both the light source and the spectrometer to illuminate the sample and collect data. All electronic devices interfaced through custom software developed in Python running on a laptop.

At each optical biopsy site, real-time monitoring was performed, including binary classification of each site on esophageal and gastric specimens, with the tissue type displayed in real time on the screen. Supervised machine learning and permutation feature importance were utilized for feature selection and performance evaluation of spectral data classification.

The results of the classification for stomach and esophagus spectral data were discussed using light gradient boosting machine (LGBM), multilayer perceptron (MLP), support vector machine (SVM), and extreme gradient boosting (XGB) classifiers. The XGB classifier demonstrated superior performance in differentiating between normal and cancerous tissues in both stomach and esophageal samples. In this validation study of 37 patients, a DRS probe with a tracking system demonstrated that machine learning can distinguish normal from cancerous tissue in real time, achieving diagnostic accuracies of 93.9% for gastric and 96.2% for esophageal specimens.

### 3.4 Real-time *in vivo* early lung cancer detection with endoscopic Raman spectroscopy

Autofluorescence bronchoscopy (AFB) combined with white light bronchoscopy (WLB) is the most precise technique for localizing lung cancers within the central airways (25, 26). However, the diagnostic accuracy of WLB+ AFB for detecting high-grade dysplasia (HGD) and carcinoma *in situ* can differ depending on the physician’s level of experience. Raman spectroscopy, which investigates molecular vibrations to provide distinct spectral fingerprint-like features, offers high accuracy for classifying tissue pathology.

**Figure 3a** shows the schematic diagram of the endoscopic laser Raman spectroscopy system. It illustrates the arrangement of the excitation and collection of fibers. A diode laser generates the Raman excitation light, which is transmitted to the tissue surface using a removable fiber optic probe inserted through the instrument channel of the bronchoscope (27). The same catheter collects emission from the tissue, which is then sent to the spectrometer for analysis. To correct spectral imaging distortion for better signal-to-noise ratio (SNR), the collection fibers are linked to the spectrograph via a unique round-to-parabolic fiber bundle.

One spectrum is captured per second, with clinical data gathered from 280 tissue sites in 80 patients (27). Multivariate analysis and waveband selection techniques on the Raman spectra are used for detecting HGD and malignant lung lesions. For spectral classification, principal components with generalized discriminant analysis (PC-GDA) and partial least squares (PLS) are used (27). Leave-one-out cross-validation (LOO-CV) is utilized, where each single spectrum is sequentially excluded for testing, and the remaining spectra are used for training. Waveband selection techniques such as stepwise multiple regression (STEP), genetic algorithm (GA), and LASSO enhance diagnostic specificity (27). For 90% sensitivity, PLS analyses with STEP waveband selection provide the best specificity of 65% (27).

Compared with WLB + AFB visual grade-3-based diagnosis, Raman +WLB + AFB improved the sensitivity of localizing HGD and malignant lesions by 1.89 times, while compared with WLB + AFB visual grade 2 + 3 combined diagnosis, Raman + WLB + AFB improved the specificity of localizing HGD and malignant lesions by 3.82 times (27). Through quantitative spectral analysis, the Raman algorithm holds promise to evolve into an automated and impartial diagnostic technique.

### 3.5 Real-time optical coherence tomography

The preresection accuracy of optical diagnosis of T1 colorectal cancer is discussed in (28). It explains large non-pedunculated colorectal polyps (LNPCPs). Endoscopists, following a standardized procedure for optical assessment, predicted the histology of LNPCPs during colonoscopy in consecutive patients. They recorded the morphological features observed under white light as well as the vascular and surface patterns identified using narrow-band imaging (NBI) (28). A multivariable mixed-effects binary logistic LASSO model was employed to develop a risk score chart (28). The reported sensitivity for the optical diagnosis of T1 colorectal cancer was 78.7%, and the specificity for optical diagnosis of endoscopic unresectable lesions was 99.0% (28). Notably, when endoscopists had low confidence compared with high confidence in their predictions, the optical diagnosis was often incorrect (28).

This suggests that considering an *en bloc* excision biopsy or consulting a trained colleague could be useful in further reducing the misidentification of T1 colorectal cancers. The ability to distinguish non-invasive LNPCPs from T1 colorectal cancers using a few white light and NBI features was quite effective (28).

Optical coherence tomography (OCT) is a potentially alternative approach to endoscopic biopsy for differentiating normal colonic mucosa from neoplasia. A deep-learning-based pattern recognition OCT system in (28) has been introduced to automate image processing. This deep-learning-based system provides accurate diagnosis, potentially in real time. This automation helps with clinical translation, which is constrained by the need to process the large volume of generated data (29). OCT, as an emerging imaging technique, obtains 3D “optical biopsies” of biological samples with high resolution. A convolutional neural network (CNN) has been employed to extract structural patterns from human colon OCT images (29). The network was trained and tested using 26,000 OCT images gathered from 20 tumor areas, 16 benign areas, and six other abnormal areas (20). Experimental diagnoses utilizing the pattern recognition (PR) OCT system was compared with established histologic findings, demonstrating a sensitivity rate of 100% and a specificity rate of 99.7%; the area under the receiver operating characteristic (ROC) curve was 0.998 (29).

**Figure 4:**
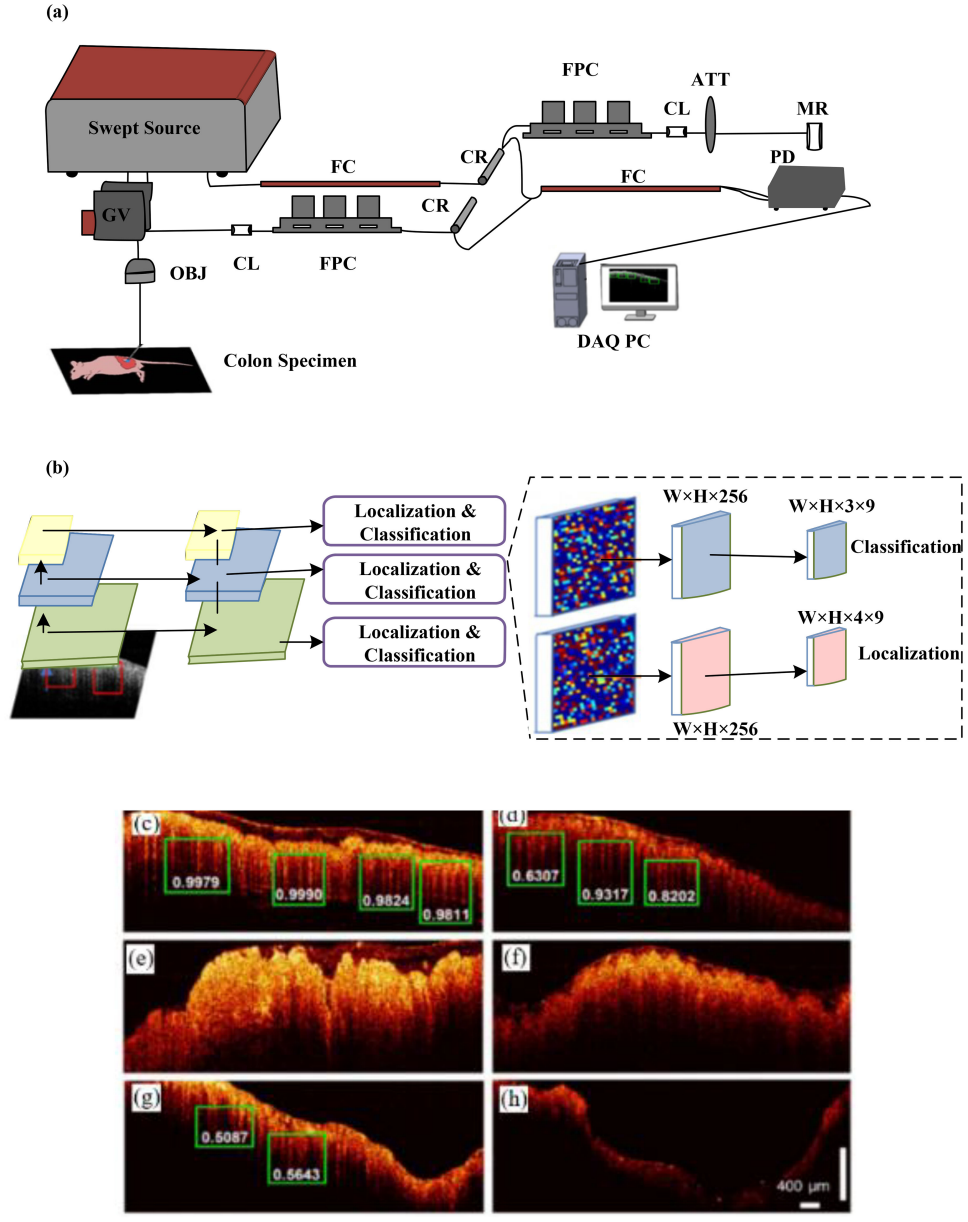
Imaging framework of pattern recognition Optical coherence tomography (PR) OCT. **(a)** Homemade SS-OCT system, where FC, CR, FPC, CL, ATT, MR, GV, OBJ, PD, and DAQ PC stand for fiber coupler, circulator, fiber polarization controller, collimator attenuator, mirror, galvo mirror system, objective lens, photodetector, and data acquisition computer, respectively (29). **(b)** RetinaNet framework through which a feature pyramid network (FPN) with a ResNet-18 backbone (left part) and two sub-networks to predict classifications and locations (right part) are shown. Pattern detection results based on PR-OCT for **(c, d)** normal colon images (green boxes indicate predicted “teeth” patterns and scores). **(e)** Colon cancer images. **(f)** Colon polyp images. **(g)** Treated complete responder colon images. **(h)** Treated non-responder colon images (29). Reproduced from (29), licensed under CC BY 4.0.

**Figure 4a** shows the swept-source OCT system. An illustration of RetinaNet is shown in **Figure 4b**, where the left portion features a feature pyramid network (FPN) built on a ResNet-18 backbone, while the right portion includes two sub-networks that handle classification and localization predictions. The input light is divided by a 50–50 fiber coupler and then guided into a reference arm and a sample arm through two circulators. Two manual fiber polarization controllers are used to control fiber polarization. A variable density filter is employed to attenuate the reference arm. A galvo mirror system is used to scan the light beam in the sample arm. The interference signal, detected by a balanced detector (PDB), is transmitted to a data acquisition board; then, the monitor displays the real-time OCT B-scan images.

For pattern recognition, the trained RetinaNet model was evaluated on the test cohort (29). Given that the “Teeth” pattern signifies normal colon specimens, the network successfully identified all “Teeth” patterns within the test OCT images (29). The PR-OCT workflow involved collecting colorectal B-scan images, separating them into training and testing sets, labeling “Teeth” and “Noise” patterns and feeding them into RetinaNet. Subsequently, the trained model was tested on the entire set of test images, followed by a performance evaluation.

**Figures 4c–h** present pattern recognition outcomes from six representative OCT images (29). In typical scenarios (**Figures 4c,d**), “Teeth” patterns are identified and highlighted with green boxes, with the respective scores displayed next to each box (29). Conversely, no such pattern is identified in the cancerous case (**Figure 4e**). **Figure 4f** illustrates the testing result for an adenomatous polyp, where no “Teeth” pattern was observed. In contrast, for treated complete responders, the “Teeth” patterns reemerge as depicted in **Figure 4g**. On the other hand, treated non-responders showed no such pattern detection (**Figure 4h**).

### 3.6 Real-time polyp detection and classification with optical tools

Utilizing AI in real-time during colonoscopy assists colonoscopists in distinguishing between neoplastic polyps that need to be removed and non-neoplastic polyps that do not require removal. A multicenter clinical study compared a computer-aided diagnosis system with standard visual inspection of small (≤5 mm in diameter) polyps in the sigmoid colon and rectum for optical diagnosis of neoplastic histology (30). The computer-aided diagnosis system employs real-time ultra-magnification to visualize polyps during colonoscopy. After making a diagnosis as to whether neoplastic, uncertain, or non-neoplastic, all polyps visualized through imaging were excised.

The study evaluated the sensitivity for neoplastic polyps achieved through computer-aided diagnosis and visual inspection compared with histopathological examination. Reference (30) suggests that using computer-aided diagnosis helped providers have higher confidence in optical diagnosis, potentially leading to cost reduction as more polyps could be left *in situ*. However, better confidence comes at a cost; computer-aided diagnosis assessment prolongs colonoscopy procedure time, which increases healthcare costs. It has been demonstrated that the time necessary for computer-aided diagnosis assessment of one small polyp is about 40 s. This is a trade-off, as computer-aided diagnosis reduces unnecessary removal of polyps and histopathologic assessment.

The standard method showed a sensitivity rate of 88.4% for detecting neoplastic polyps, compared with 90.4% using the computer-aided diagnosis (CADx) approach. The two methods differed in 7.2% of cases. Specificity was 83.1% for the standard method and 85.9% for CADx, with a 7.9% rate of disagreement between them. The percentage of polyp assessments with high confidence for categorization into neoplastic or non-neoplastic polyp increased from 74.2% with the standard method to 92.6% with the CADx method. For small polyps during colonoscopy, AI polyp detection tools known as computer-aided polyp detection provide potential detection by up to 50%.

## 4 Real-time elastography and mechanical imaging

This section describes different elastography procedures, starting with real-time shear-wave elastography. Subsequently, it elucidates the significance of real-time elastography (RTE)-guided biopsy in prostate cancer detection and diagnosis.

### 4.1 Real-time shear-wave elastography

In (31), two statistical analyses were performed to assess the effectiveness of real-time US shear-wave elastography (SWE) in identifying and pinpointing malignant prostatic lesions using both a patient-based and a region of interest (ROI) or sextant-based approach. The patient-centered approach accounts for the possibility of multiple findings within the same patient and their specific locations—for instance, a patient with a single false-positive lesion is managed in the same way as a patient with multiple false-positive lesions. This leads to variations in both the numerator and denominator of the false-positive rate compared with those in the sextant evaluation. Additionally, patients may present with both true-positive and false-positive findings. This accounts for the reduced diagnostic performance of SWE observed in the per-patient analysis (31). The 95% confidence intervals for ROI-based estimates were consistently narrower than those for patient-based estimates in nearly all cases. In general, the sextant-based approach is more effective than the patient-based approach, as it allows clinical treatment to be tailored to each individual sextant. The number of required biopsies can be decreased with a negative result in an SWE examination.

The effectiveness of RTE-targeted biopsy for detecting and diagnosing prostate cancer remains uncertain (31). In reference (30), the diagnostic accuracy of RTE-targeted biopsy was evaluated using relative sensitivity, with the 10-core systematic biopsy serving as the reference standard. The analysis included seven studies—five cohort studies and two randomized controlled trials (RCTs). Across the five cohorts involving 698 patients, RTE-targeted biopsy did not surpass the systematic approach in overall prostate cancer detection (69.5% vs. 80.5%, relative sensitivity = 0.92) or in initial biopsies (56.8% vs. 64.0%, relative sensitivity = 0.93). However, in the core-by-core comparison, RTE-targeted biopsy identified a higher proportion of positive cores than systematic biopsy (21% vs. 11%, relative sensitivity = 2.17). The two RCTs indicated a trend toward improved detection when RTE-targeted biopsy was combined with systematic biopsy compared with the systematic method alone (45.5% vs. 39.5%, risk ratio = 1.18).

**Figure 5:**
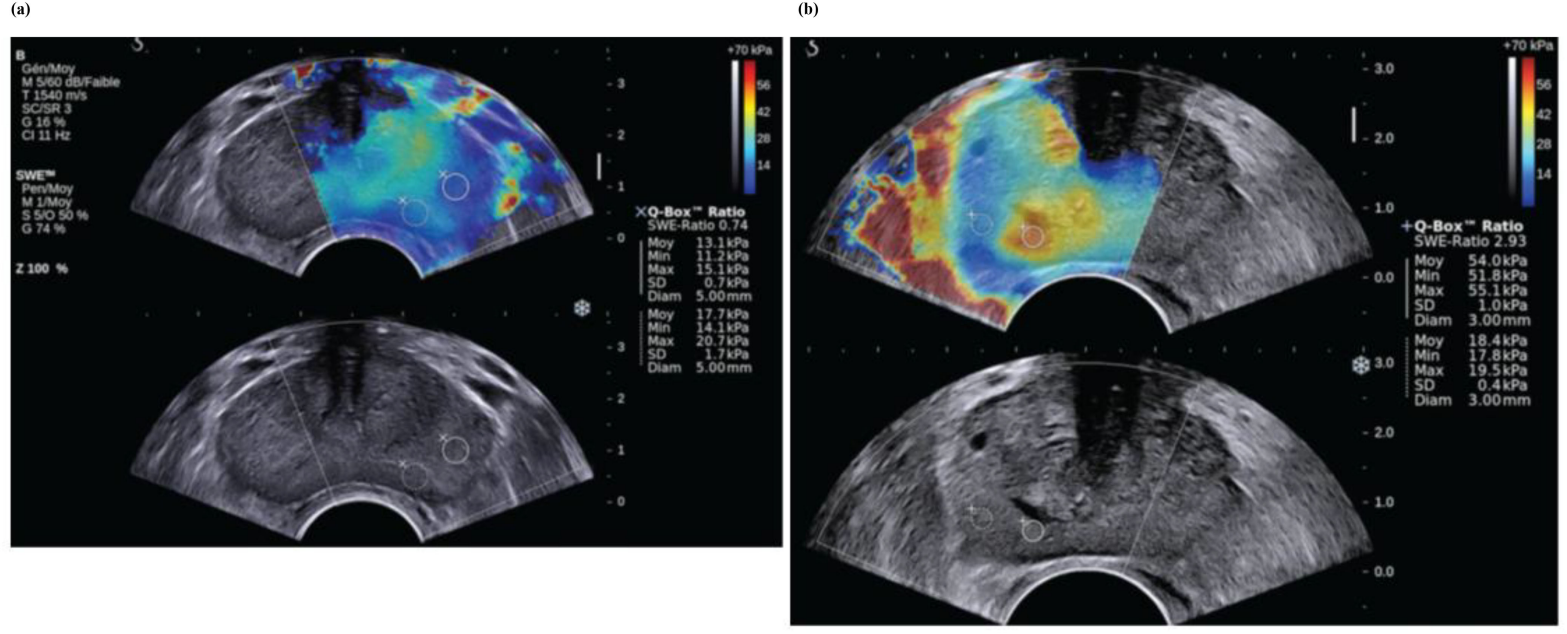
**(a)** Shear wave elastography (SWE) prostate measurements in a 58-year-old male patient (with a prostate-specific antigen (PSA) level of 4.6 ng/mL). The upper image displays the color-coded SWE (within the color box). In the lower image, only B-mode data for the same plane is shown. **(b)** SWE prostate measurements in a 57-year-old man (with a PSA level of 6.62 ng/mL). An orange-coded stiff area (incidentally found in the left base paramedian) is shown in the upper image, while in the lower image, a peripheral zone with no abnormal features on B-mode US is shown [from (31)]. Reproduced with permission from (31), © Radiological Society of North America (RSNA).

To do SWE acquisition, the following steps are required: The elastographic box was maximized to cover half of the gland in a transverse plane. Each side of the prostate’s peripheral zone was scanned separately from base to apex with a very slow motion to stabilize the signals. The two digital cine loops were saved for subsequent stiffness measurements. After completing the SWE acquisition, each digital cine loop was reviewed. Systematic stiffness measurements were conducted at the site of maximum stiffness within each sextant of the peripheral zone using a ROI shown in **Figure 5a**. It shows a SWE prostate measurement in a 58-year-old man with a PSA level of 4.6 ng/mL (31). The top image displays the color-coded SWE within the color box. The bottom image illustrates the same plane with only B-mode information. Mean elasticity values in SWE were measured by placing two circular ROIs in the paramedian and lateral sextants (31). The ROI was positioned on the peripheral zone using the B-mode image, where the peripheral zone was distinctly visible (shown in **Figure 5b**). It shows a SWE prostate measurement in a 57-year-old man with a PSA level of 6.62 ng/mL (31). The top image displays an orange-coded stiff area (solid-line ROI) incidentally found in the left base paramedian peripheral zone, which shows no abnormal features on the B-mode ultrasound (bottom image) (31).

The ROI, indicated by a dashed line, was situated in the lateral peripheral zone of the same sextant. The biopsy from the stiffest area revealed an 11-mm Gleason 7 adenocarcinoma (31).

### 4.2 Real-time video-based invasion depth assessment

Diagnosing the invasion depth of colorectal cancer using white light (WL) and image-enhanced endoscopy (IEE) techniques remains a significant challenge (32). To determine the invasion depth of colorectal cancer, a dual-modal deep learning-based system, incorporating both WL and IEE, has been constructed and validated (32). This approach combines features from both WL and IEE images for AI-based assessment of invasion depth in colorectal cancer (32).

WL and IEE images were paired together for analysis. For a real-time evaluation of the endo-colorectal cancer system performance, 35 videos were used. Two deep learning models were developed: one using WL images (referred to as model W) and another using IEE images (referred to as model I). These models were compared with the endo-colorectal cancer system.

WL and IEE images are used in a CNN-based system for the diagnosis of colorectal cancer invasion depth (32). This system was developed and evaluated across three hospitals, demonstrating its ability to detect lesions unsuitable for endoscopic resection in independent test images with high accuracy.

**Figure 6:**
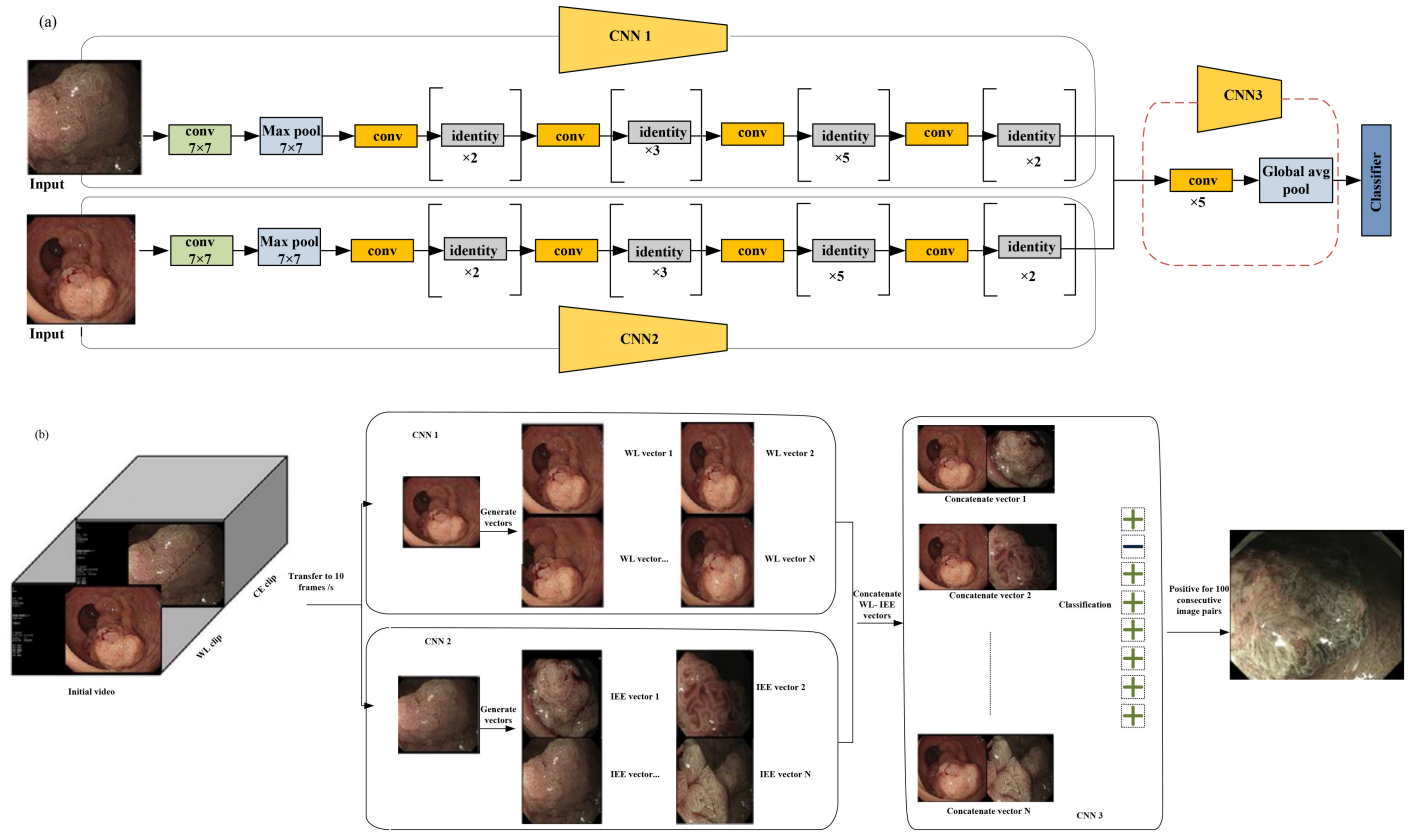
**(a)** Multimodal data used for training in endo-colorectal cancer. White-light (WL) images and image-enhanced endoscopy (IEE) images are paired (with each pair containing one WL image and one IEE image). To obtain eigenvectors from the WL and IEE images, pretrained convolutional neural network (CNN) models (CNN1 and CNN2) based on ResNet-50 were employed, respectively. Feature maps were generated from concatenated vectors (vector-IEE concatenated with vector-WL) to be used in classifying a self-constructed CNN3. **(b)** Workflow of video testing. with a rate of 10 frames per second; the videos were converted to images. Each IEE vector was combined with every WL vector sequentially. An unsuit-ER result was generated when 100 consecutive image pairs were classified as positive; otherwise, a suit-ER result was produced. “Suit-ER” and “unsuit-ER” stand for lesions suitable and unsuitable for endoscopic resection, respectively [adapted from (32)]. Reproduced from (32) (Gastrointestinal Endoscopy), with permission from Elsevier.

In **Figure 6a**, three models were developed for comparison of the performance of WL images, IEE images, and the combination of WL and IEE images in diagnosing CRC invasion depth. These models are W, I, and Endo-CRC.

The WL image entered the WL convolution network, and the IEE image entered the IEE convolution network; then, for feature concatenation, a convolution block was used (**Figure 6a**).

Two pretrained CNN1 and CNN2 models are based on ResNet-50. These models were used to obtain eigenvectors from the WL and IEE images. In each image pair, the IEE vector was concatenated with the WL vector, and the resulting combined vector was used to generate feature maps. Finally, these feature maps were used to classify a self-constructed CNN3 (32). To evaluate the clinical applicability of the endo-CRC system, video clips were used as the validation dataset.

These videos were 10 to 19 s. As previously mentioned, the endo-CRC system diagnoses invasion depth using image pairs of lesions. Consequently, the endo-CRC system remains inactive and stores WL images at a rate of 10 frames per second (fps) during the WL clip (32).

When the video switches to IEE mode, each IEE image frame is paired with all WL images. The system then processes each image pair sequentially, outputting the results accordingly. A lesion was deemed unsuitable for endoscopic resection if 100 consecutive image pairs were classified as positive in each video. Otherwise, the lesion was considered suitable for endoscopic resection (suit-ER) (**Figure 6b**). The system achieved a processing speed of 45 frames per second (fps) on the GPU. This processing speed fulfills the real-time processing speed requirement of 25 fps for endoscopic videos. Each video was converted to images at a rate of 10 frames per second. Subsequently, each IEE vector was sequentially combined with all WL vectors. If 100 consecutive image pairs were classified as positive, the system determined the lesions to be unsuitable for endoscopic resection (unsuit-ER). Otherwise, the lesions were deemed suitable for endoscopic resection (suit-ER). Endo-CRC demonstrated accuracies of 91.61% and 93.78% in internal image tests with and without advanced colorectal cancer (CRC), respectively, and 88.65% in the external test, which excluded advanced CRC cases. In a head-to-head comparison with endoscopists, Endo-CRC achieved an expert-level accuracy of 88.11% and showed the highest sensitivity among all participants. In video-based evaluation, its accuracy reached 100%. When compared with other models, Endo-CRC performed better, with higher accuracy per image pair (91.61% vs. 88.27% for model I and 91.61% vs. 81.32% for model W).

## 5 Real-time neuromorphic AI systems for cervical cancer diagnosis

**Figure 7:**
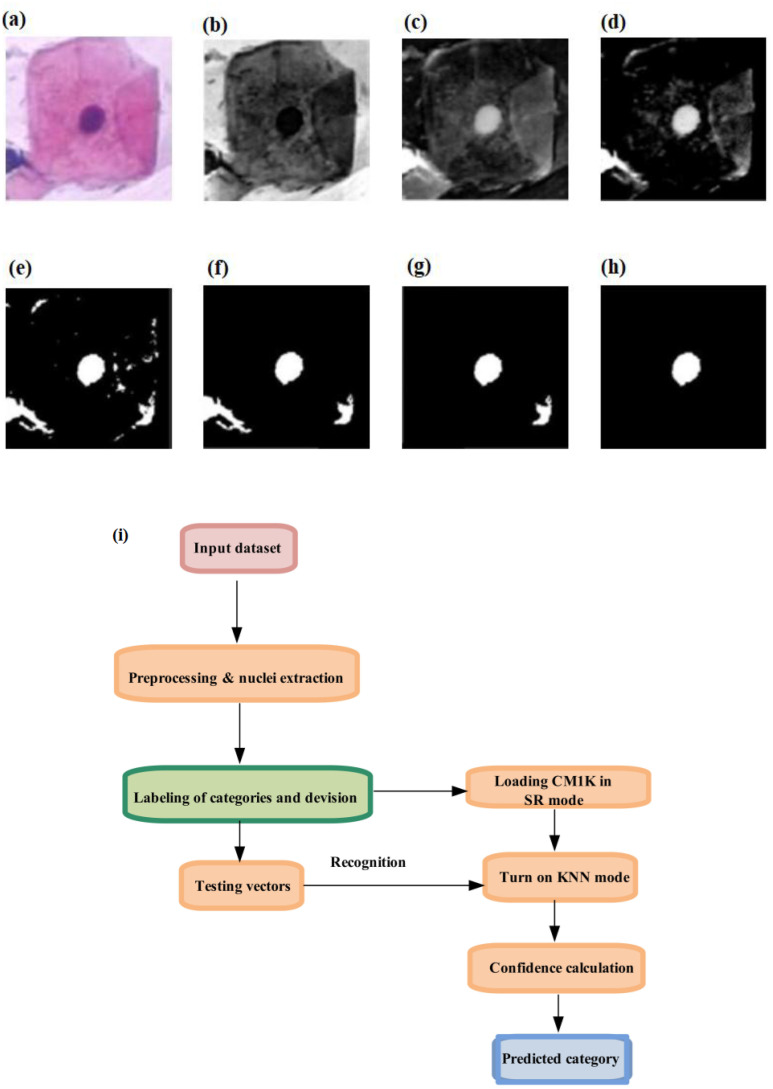
Pre-processing steps and hardware processing flow: **(a–h)** original image, top-hat filtered, bottom-hat filtered, subtracted image, binarized image, post noise-removal, segmented image, and detected nucleus, respectively (33). **(i)** Flow for the hardware accelerator technique containing learning, recognition, and classification [from (33)]. Reproduced from (33) (IEEE BioCAS Conference), with permission from IEEE.

An ultra-fast, low-power dedicated neuromorphic hardware for intelligent classification to detect cervical cancer via utilizing single-cell and multi-cell images has been introduced (33). This neuromorphic hardware employs the bio-inspired ASIC CM1K Neuromem chip (33). The approach was evaluated using both single-cell and multi-cell images, with hardware performance benchmarked for accuracy, latency, and power consumption. These metrics were compared against traditional software implementations, including K-nearest neighbor (KNN) and support vector machine (SVM). **Figures 7a–d** illustrate the preprocessing steps applied to real dataset images. Preprocessing was carried out on an Intel i5 6th gen PC. The average preprocessing times were 335.5 ms for normal single-cell images and 136.3 ms for abnormal single-cell images. For multi-cell images, the average times were 728.6 ms for normal and 737.9 ms for abnormal images. **Figures 7e–h** display the original image, top-hat filtered image, bottom-hat filtered image, subtracted image, binarized image, post noise-removal image, segmented image, and detected nucleus (33).

**Figure 7i** depicts the complete process of learning, recognition, and classification within the diagnostic system. The nuclei area values were normalized to a range of 0 to 255 to ensure the system’s independence from magnification. This normalization allowed the classification to be based on the relative sizes of the nuclei within the images. For each image used in training the database, one neuron was assigned, with the area metrics encoded into a byte array vector. This vector was then labeled with a category, either normal or abnormal, and loaded onto the neural network. The on-chip recognition was performed using the KNN mode. Testing vectors were broadcast for recognition one at a time. For single-cell images, the accuracy was comparable to and, in some cases, surpassed that of KNN and SVM classifiers. The Neuromem chip can be trained in two ways: *by directly loading saved knowledge onto the chip (save-*and-restore (SR) mode) or by enabling the neurons to learn in real time and build a knowledge base dynamically (normal mode).

In SR mode, training each neuron on the Neuromem chip took approximately 18 μs, compared with a few milliseconds for KNN and SVM classifiers. The hardware-based recognition time/per vector does not rely on the size of the training dataset. In comparison to traditional KNN/SVM implementations, the average speed-up in training time per vector is approximately 596 times for single-cell images and 477 times for multi-cell images. The average speed-ups in recognition time per vector for single-cell and multi-cell images are approximately 54 times and 41 times, respectively, compared with conventional methods.

For validation using both single- and multi-cell images, the hardware implementation achieved a maximum mean accuracy of 90% for single-cell and 86.67% for multi-cell analyses. The average performance showed a recognition time speed-up of approximately 54× for single-cell and 41× for multi-cell processing. This work is limited by its reliance on relatively basic preprocessing techniques and the use of a single feature for the two-level classification of normal and abnormal samples. Enhancing the hardware approach with sophisticated preprocessing techniques, such as those achieved with deep learning (34) or GPUs, along with the utilization of multiple features (35), will significantly enhance the accuracy of the diagnostic system. Secondly, while the dataset for multi-cell images was small, the system’s performance matching that of software implementations suggests that it could achieve similar results with larger and more varied datasets.

## 6 Real-time fluorescence imaging and optical navigation

This section explains various fluorescence-imaging-based methods for cancer diagnosis. It discusses real-time fluorescence image guidance based on a goggle-augmented imaging as well as navigation system. Additionally, it covers breast cancer diagnosis through pH-based fluorescence lifetime endoscopy and examines the use of fluorescence lifetime imaging to delineate tumor margins in excised breast tissue samples.

### 6.1 Goggle augmented imaging and navigation system

In (36), for tumor resection, a goggle augmented imaging and navigation system (GAINS) has been introduced. The system has shown high accuracy in detecting tumors in subcutaneous and metastatic mouse models. Human pilot studies involving breast cancer and melanoma patients have shown that GAINS can identify sentinel lymph nodes with perfect accuracy, achieving 100% sensitivity when using a near-infrared dye (36). The clinical application of GAINS for guiding tumor resection and mapping sentinel lymph nodes holds significant potential for enhancing surgical outcomes. It aims to reduce the likelihood of repeat surgeries and improve the precision of cancer staging, thereby offering a more effective approach to cancer treatment.

**Figure 8:**
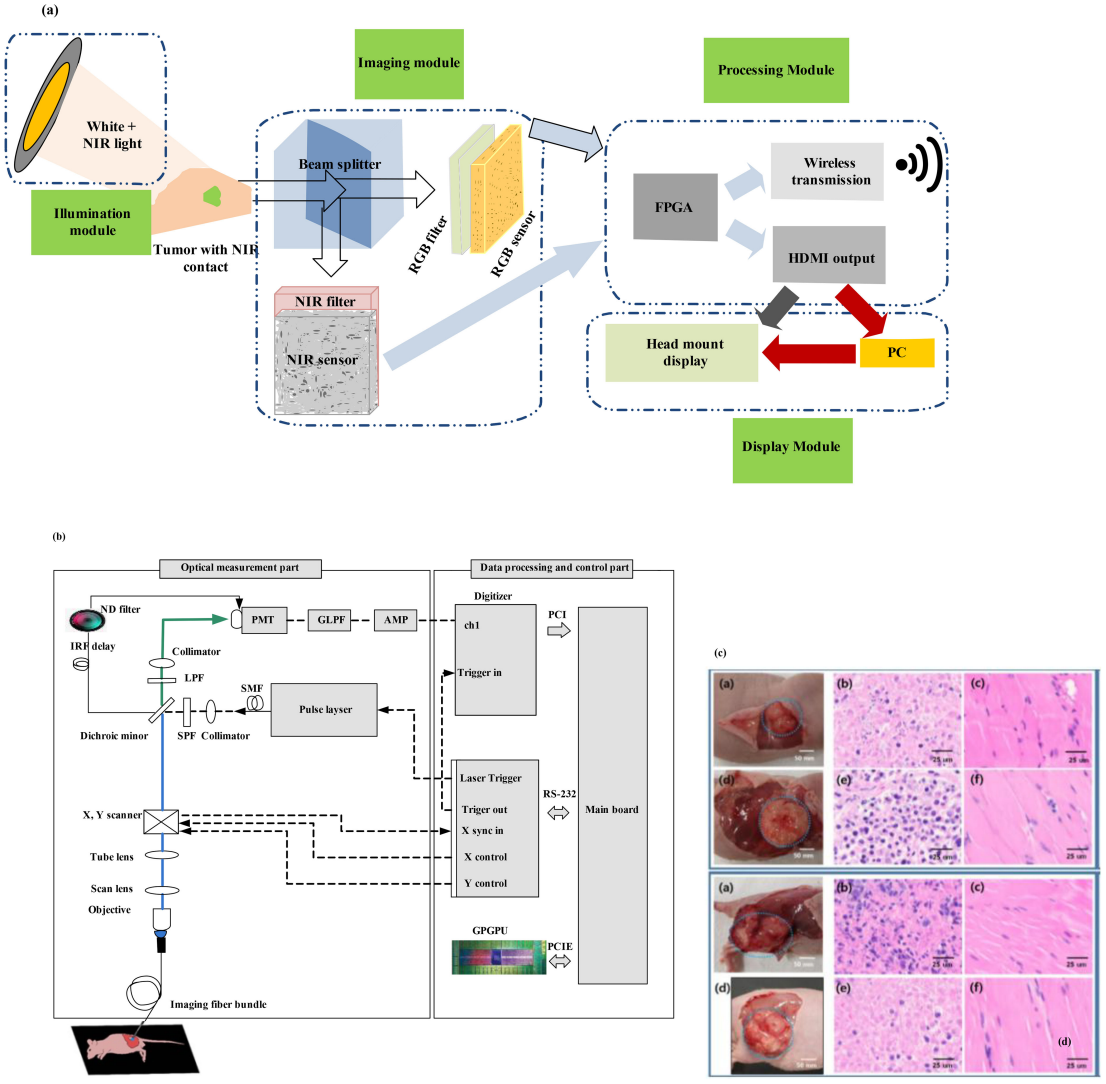
**(a)** Framework of the goggle augmented imaging and navigation system (GAINS) system with its different modules and information flow between the modules (36). Reproduced from (36), licensed under CC BY 4.0. **(b)** Schematic of the experimental setup for fluorescence lifetime endoscopy (FLE) (37). **(c)** Images at the top show the breast cancer model, and those at the bottom show the skin cancer model. **(b)** H&E staining results of tissues from the normal group and **(c)** from the tumor group [adapted from (37)]. Reproduced from (37), licensed under CC BY 4.0.

**Figure 8a** illustrates the design of GAINS. The processing unit generates co-registered composite color-fluorescence images, which are displayed in real time via a lightweight and high-resolution head-mounted display (HMD) unit. The near-infrared (NIR) source is composed of light-emitting diodes (LEDs) paired with a bandpass filter. The display module features a high-resolution HMD. To align the imaging module with the camera’s weight and the user’s field of view (FOV), an adjustable and counterbalanced mechanical mounting system was integrated (36). This system can improve user experience. As the concentrations of NIR contrast agents increase, GAINS determines the fluorescence intensity profile. To test GAINS functionality *in vivo*, a subcutaneous breast cancer mouse model was used. Utilizing this image processing algorithm generates in real time composite color-fluorescence images.

These images are concurrently displayed on both an HMD and a nearby personal computer. This system enables non-intrusive information display to the operating surgeon while simultaneously making the data available to the entire surgical team in the operating room. Overlaying fluorescence information on the normal visual field can facilitate rapid intraoperative tumor visualization. However, a larger sample size is necessary to confirm these results. Statistical analysis was conducted using OriginPro8. Paired *t*-tests were employed to compare fluorescence signals between tumors and background tissue in mouse models. The sensitivity of sentinel lymph node (SLN) detection was compared among the GAINS method, radioactivity, and blue dye techniques. These measurements showcase non-intrusive real-time image guidance. The minimal training needed has the potential to make this technology more accessible to clinicians.

Using tumor-targeted contrast agents, the system accurately detected tumors in both subcutaneous and metastatic mouse models, achieving 100% sensitivity and 98% ± 5% specificity. In preliminary human studies involving breast cancer and melanoma patients, application of a near-infrared dye enabled the GAINS system to identify sentinel lymph nodes with 100% sensitivity. Clinically, GAINS-guided tumor resection and lymph node mapping have the potential to enhance surgical precision, lower repeat surgery rates, and improve the accuracy of cancer staging.

### 6.2 Real-time cancer diagnosis using pH-based fluorescence lifetime endoscopy

A biopsy is often conducted during surgical procedures for cancer diagnosis. Pathological biopsy of surgical specimens is also necessary to differentiate cancerous tissues from normal tissues. In (37), a novel method using fluorescence lifetime endoscopy (FLE) has been employed to discriminate between tumors and normal tissues (**Figure 8b**). Reference (37) discusses the feasibility of real-time, *in vivo*, and *in situ* cancer diagnosis. By analyzing fluorescence lifetime data in tissues, the need for tissue resection is eliminated. This study has been demonstrated in 20 mice, including two different types of mouse models with breast and skin cancer. The pH-related fluorescence lifetime results for normal and tumor tissues have been validated, showing consistency with the outcomes of the H&E staining test (37).

These findings are promising for intraoperative laparoscopic surgery applications, enabling the assessment of cancer using an *in vivo* FLE system. However, for clinical applications, it will be necessary to perform pH assessment *in vivo* in tissues as an optical biopsy. Cancer diagnosis based on fluorescence lifetime has also been suggested for topical applications that reduce tissue absorption and fluorophore penetration. Fluorescein has been approved for clinical use through intravenous administration. In **Figure 8b**, the objective lens functions as an integral component, bridging the scan lens and the imaging fiber bundle system to form the endoscope system. The imaging fiber bundle system comprises a fiber bundle and an imaging lens, with the imaging bundle positioned at the focal point of the objective lens. The laser beam is directed through the scan lens into the imaging fiber bundle system to excite the tissue. The PMT, which is connected to the digitizer, collected the reflected emission beam; this enables acquisition of 2D fluorescence lifetime information of tissues. This system can be utilized in gastroenterology by inserting the imaging fiber bundle into the biopsy channel of a standard commercial endoscope. **Figure 8c** shows images of the breast cancer model (top) and skin cancer model (bottom). The H&E staining results of tissues are shown in **Figure 8c**, wherein columns 2 and 3 display the results from the normal group and the tumor group, respectively. The fluorescence lifetime endoscopy system distinguished normal from breast tumor tissue with high accuracy, showing mean lifetimes of 5.96 ns (normal) versus 4.60 ns (tumor; *p* < 0.0001). The method achieved real-time, pH-dependent cancer detection sensitivity of about 0.06 ns per pH unit, demonstrating a clear contrast between malignant and healthy tissues.

Tissues from the tumor group showed cell and nuclear damage compared with those from the normal group. The presence of tumor tissues over the evaluated regions of the tissue was validated using this technique. Cancer cells exhibited larger size, giant nuclei, dual nuclei, multiple nuclei, or irregular nuclei and a different nucleus-to-cytoplasm ratio compared with normal cells (37).

Using fluorescence lifetime imaging for detecting tumor margins in excised breast specimens in breast-conserving surgery, ensuring tumor-free surgical margins is crucial. However, in as many as 38% of cases, a second surgery becomes necessary because malignant cells are detected at the edges of the excised resection specimen.

Consequently, sophisticated imaging tools are essential to guarantee clear margins during surgical procedures. A random forest classifier has been investigated to diagnose tumors at resection margins. This provides an intuitive visualization of probabilistic classification on tissue specimens.

Fluorescence lifetime imaging (FLIm) measurements is used in this classifier. FLIm uses parameters derived from point-scanning label-free of breast specimens. FLIm data obtained from fresh lumpectomy and mastectomy specimens were used (38). A registration technique between autofluorescence imaging data and cross-sectional histology slides is used to conduct the supervised training (38). This approach offers high prediction accuracy, swift acquisition speed for large-area scans, spatial refinement for suspicious regions, and nondestructive probing (38). This method offers intuitive visualization of tissue characteristics and achieves high tumor classification sensitivity and specificity of 89% and 93%, respectively.

**Figure 9:**
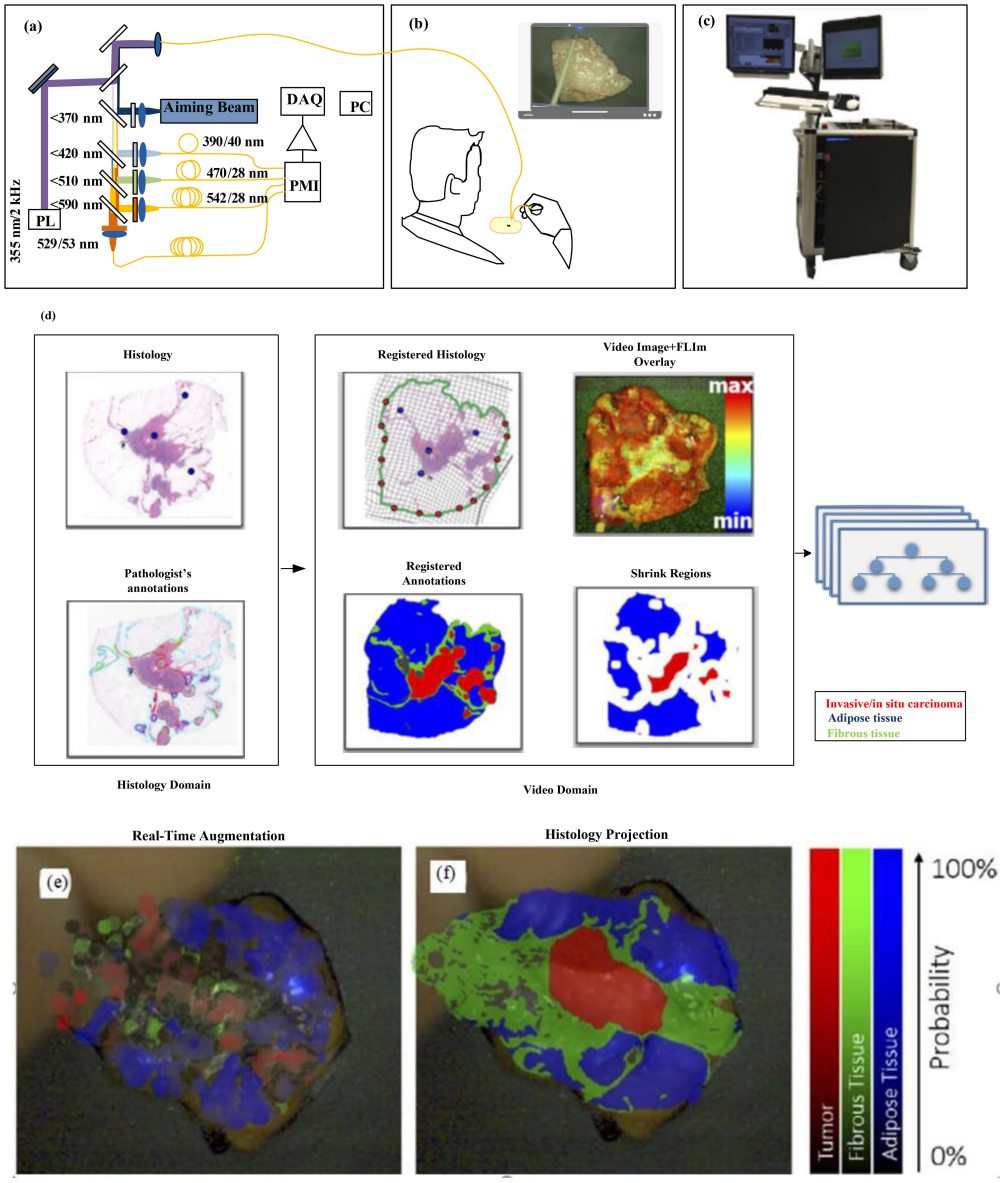
**(a)** The ms-TRFS imaging framework. For excitation and autofluorescence collection, a single fiber is used. PL, DAQ, and PMT represent pulsed laser, data acquisition, and photomultiplier, respectively (38). **(b)** Imaging setup is done as follows: A manual scan was conducted for each specimen. In order to overlay fluorescence data onto the video at the measurement site, an aiming beam embedded in the optical path was used as a marker. **(c)** The FLIm system and computers were configured into a setup (that included two screens) to image the breast specimens (38). **(d)** A supervised pipeline used for training in which for registration it uses cross-sectional histology and the video image. Within this method, the pathologist tracings were translated from the histology to the video domain, and a random forest classifier is used for classification, fed by fluorescence parameters from the resulting regions. **(e, f)** For invasive carcinoma, augmented overlay with histological ground truth reveals a marked decrease in prediction accuracy, with an erroneous classification of tumor through the overlay (38). Reproduced from (38), licensed under CC BY 4.0.

**Figure 10:**
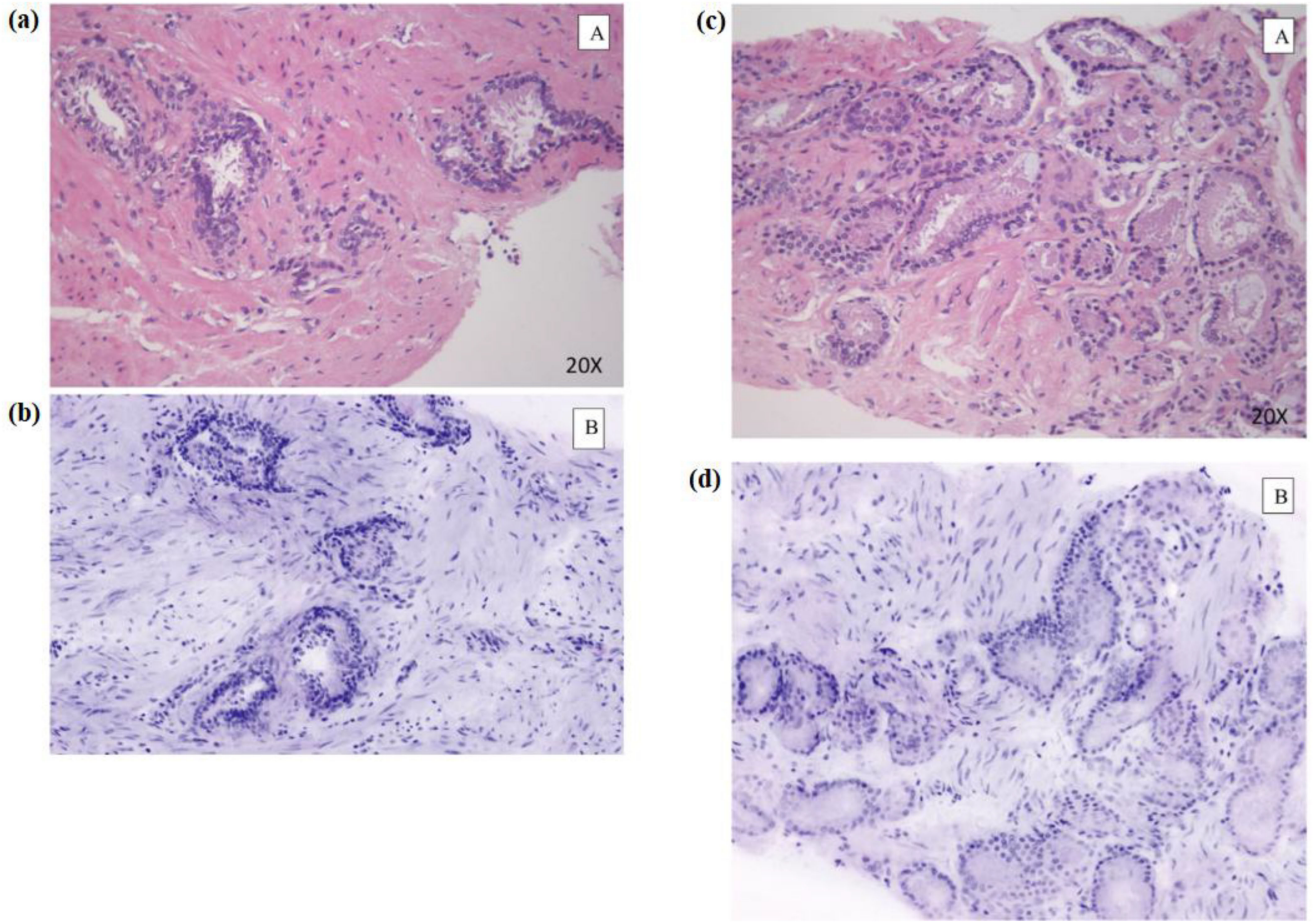
Noncancerous prostate tissue in **(a)** HE and **(b)** FCM. Cancerous prostate glands in **(c)** HE and **(d)** FCM. FCM and HE stand for fluorescence confocal microscopy and hematoxylin–eosin, respectively [adapted from (39)]. Reproduced from (39), licensed under CC BY 4.0.

**Figure 9a** displays a prototype time-domain multispectral time-resolved fluorescence spectroscopy (ms-TRFS) system featuring an integrated aiming beam (38). During imaging, the fiber probe was manually guided (**Figure 9b**) for all specimens. Throughout the scanning process and during the scanning process, the distance between the sample and the probe was maintained at a few millimeters. When the fiber tip strayed too far from the sample, the computer detected a drop in signal amplitude and triggered an acoustic alarm to alert the operator to reposition closer to the sample. An aiming beam acting as a marker to superimpose fluorescence data onto the video is integrated into the optical path. The FLIm system and associated computers are mounted on a cart, with dual screens as shown in **Figure 10c**, to image the breast specimens.

**Figure 9d** shows the training pipeline. A semiautomatic histology registration procedure was used to map the histology slide and the pathologist’s annotations onto the video image (38). Pathologist tracings from the histology are transferred to the video domain. Fluorescence parameters from the resulting regions are then sent to a random forest classifier. The output color is used to encode the type of tissue in color, and saturation represents the certainty of the probabilistic output. **Figure 9f** displays the overall accuracy which varies the percentage of training samples in 2% increments, ranging from 2% to 100%. When over 75% of the training samples are utilized, the results stabilize, achieving an overall accuracy of approximately 97.5%.

### 6.3 Real-time digital biopsy tools

Microscopic analysis of tissue is the definitive method for detecting cancer (39). Conventionally, the reporting of prostate biopsies using hematoxylin and eosin (H&E) staining involves fixation, processing, the preparation of glass slides, and analysis using an analog microscope by a local pathologist (39). Real-time remote access and image digitalization are employed to streamline the reporting process and provide a foundation for AI and machine learning.

A new optical technology named fluorescence confocal microscopy (FCM) allows immediate digital image acquisition like H&E staining, eliminating the need for conventional processing methods. Prostate biopsies were conducted at a single coordinating unit to assess consecutive patients (based on clinical indications) (39). FCM digital images obtained immediately from prostate biopsies were stored; then, after applying conventional H&E processing on the glass slides, they were digitized and stored.

**Figure 10** shows noncancerous prostate tissue in (a) H&E and (b) FCM and cancerous prostate glands in (c) H&E and (d) FCM. The minimum sample size was determined based on the accuracy of sensitivity or specificity estimates in diagnostic test studies. This calculation ensures that the sample size is sufficient for estimating inter-rater agreement using Cohen’s kappa, with specified confidence limits reflecting the accuracy of sensitivity or specificity estimates. FCM with VivaScope has the potential to elevate microscopic analysis in real time. VivaScope is compact and space-efficient, making it easily adaptable for use in the operating room. Moreover, acquiring digital images is straightforward and does not necessitate specific technical skills. A total of 854 images were analyzed by each pathologist. Prostate cancer (PCa) detection using FCM showed strong concordance with the final H&E diagnoses, achieving 95.1% accuracy (Cohen’s kappa coefficient (*κ*) = 0.84). The inter-pathologist agreement for PCa detection was nearly perfect for both H&E (agreement = 0.98, *κ* = 0.95) and FCM (agreement = 0.95, *κ* = 0.86). However, concordance for cancer grading was only moderate in both methods (H&E, *κ* = 0.47; FCM, *κ* = 0.49).

## 7 Real-time hyperspectral-imaging-based cancer diagnosis

In this section, different hyperspectral imaging techniques will be discussed. First, VNIR–NIR hyperspectral image fusion for brain cancer detection and *in vivo* skin cancer assessment via millimeter-wave-based imaging are discussed. Next, for brain cancer detection during surgical operations, using hyperspectral images with spatio-spectral classification is discussed. After that, hyperspectral-imaging-based intraoperative visualization systems are discussed. Then, *in vivo* identification of glioblastoma tumors based on hyperspectral images and employing a deep learning framework is discussed. Finally, YOLO-based hyperspectral imaging for esophageal cancer diagnosis is explained.

### 7.1 Real-time VNIR–NIR HSI fusion

For brain tumor resection assistance during surgery, it is observed that intraoperative guidance tools have limitations (40). During surgery in real time, to delineate brain tumor tissue, hyperspectral imaging offers new advancements. However, hyperspectral acquisition systems face limitations in spatial and spectral resolution. To improve spatial and spectral resolution, image fusion combines information from different sensors.

**Figure 11:**
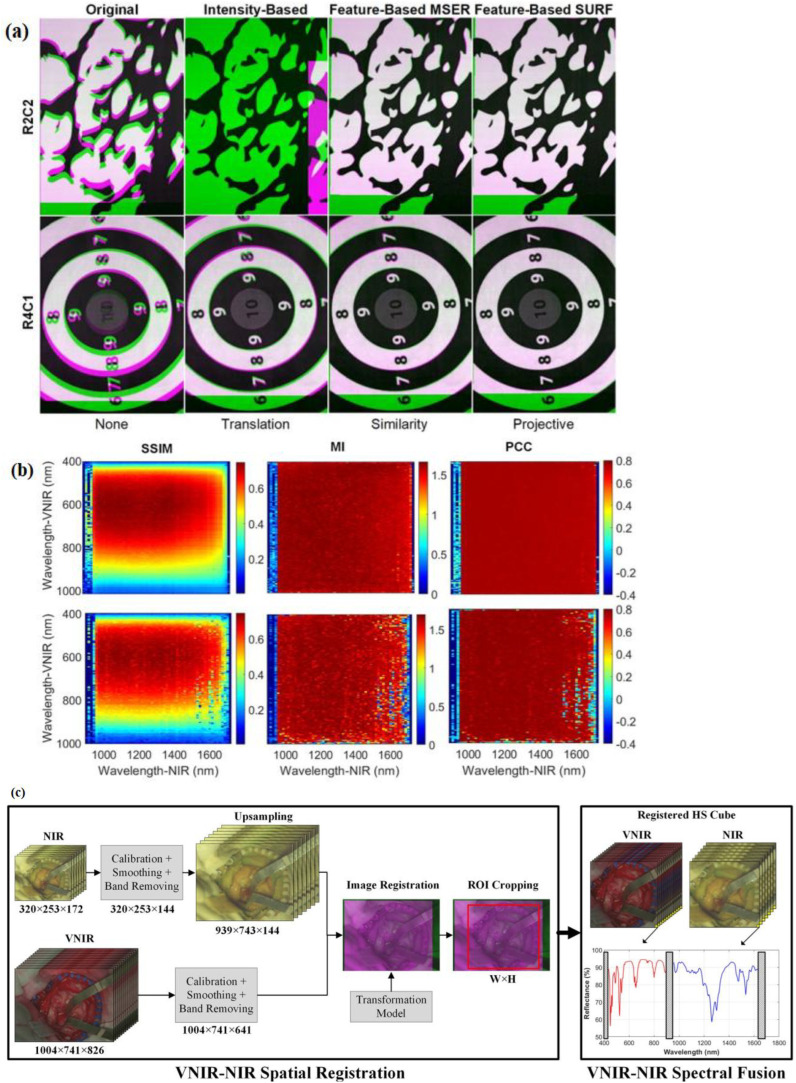
**(a)** Spatial registration process of VNIR–NIR (40). Different registration techniques are applied to two registration result examples (R2C2 and R4C1). For image overlap visualization, VNIR is in green and NIR is in magenta. The default registration is shown in the first column without applying any transformation to the data. The second column shows intensity-based results, the third column shows feature-based with MSER, and feature-based with SURF techniques are shown in the fourth columns. **(b)** Identification of the suitable spectral bands for registration (via using the feature-based SURF technique with projective transformation) is performed with coarse search results of the structural similarity index measure (SSIM), mutual information (MI), and Pearson’s correlation coefficient (PCC) (40). **(c)** VNIR–NIR spatial registration combined with spectral fusion flow diagram of the processing framework [adapted from (40)]. Reproduced from (40), licensed under CC BY 4.0.

In (40), an intraoperative hyperspectral acquisition system for image fusion uses two push-broom hyperspectral cameras covering the visual and near-infrared (VNIR) (400–1,000 nm) and near-infrared (NIR) (900–1,700 nm) spectral ranges. To obtain a hyperspectral cube with a wide spectral range (435–1,638 nm), both hyperspectral images were registered using intensity-based and feature-based techniques. For the hyperspectral imaging registration dataset, R2C2 and R4C1 are provided as two examples, as shown in **Figure 11a**. The initial column presents the registration result without any geometric transformation. The following columns display the optimal outcomes from each registration method and their corresponding best geometric transformations. Green-magenta false-color images overlay the VNIR and NIR pseudo-RGB images. Misregistration between the VNIR and NIR images are shown with magenta and green pixels, while gray-scale pixels indicate similar intensity values in the two registered images.

In the intensity-based registration employing the translation transformation, R2C2 is incorrectly registered, and compared with the outcome without employing any transformation, R4C1 improves the registration. The reason for these incorrect registrations is the random noise that is found in some spectral bands. This random noise affects the maximum intensity. The feature-based maximally stable extremal regions (MSER) approach, combined with similarity transformation, enhances the effectiveness compared with traditional intensity-based methods, though in both images some misregistered pixels can be observed. Using projective transformation, the feature-based speeded-up robust features (SURF) technique had the best results. A coarse-to-fine search was applied to identify the VNIR and NIR bands. This was achieved using grayscale images from a single spectral band captured by both cameras.

**Figure 11b** shows the R2C2 and R4C1 heatmaps resulting from the coarse search using the similarity index measure (SSIM), mutual information (MI), and Pearson’s correlation coefficient (PCC) metrics (40). Due to considering image structure, the SSIM metric provides the highest results, showing the regions 500–700 and 950–1,500 nm in the VNIR and NIR ranges, respectively (40). The other metrics only consider image intensity.

In the intensity-based registration employing the translation transformation, R2C2 is misaligned. Compared with the result, without using segmentation maps obtained with the K-means algorithm, R4C1 improves registration (40). For every hyperspectral image, the Jaccard metric was calculated based on the ground-truth image and the segmentation map. The VNIR data exhibited superior performance in color segmentation when analyzed with the K-means algorithm. This was followed by the fused data, which showed improved segmentation results using the K-medoids and hierarchical K-means (HKM) algorithms. Material identification performed exceptionally well using NIR data across all three algorithms. The application of HKM to NIR data led to significant improvements in material-color segmentation, while additional enhancements were observed in the fused data processed with K-means.

Statistical analysis of the segmentation results was conducted using a paired, one-tailed Student’s *t*-test with a significance level of 5%. The proposed approach in (40) uses a two-step method which is composed of VNIR–NIR spatial registration and VNIR–NIR spectral fusion (**Figure 11c**).

The raw VNIR and NIR images undergo image calibration to counteract environmental illumination effects, noise filtering, and band removal processing steps. Then, to enable image registration, the VNIR pixel size has been matched to the NIR image via upsampling. This upsampling is achieved via employing a generated transformation model, in which the fixed image is the VNIR and the moving image is the NIR.

To obtain the same ROI, after registration of VNIR and NIR images, they are cropped. Finally, in the last stage, the spectra from both VNIR and NIR images are combined. To facilitate spectral fusion, a reflectance offset has been applied to the NIR spectrum. By combining VNIR and NIR data, classification accuracy improved by up to 21% compared with using either modality alone.

### 7.2 Real-time *in vivo* skin cancer detection with millimeter−wave imaging

High-resolution millimeter-wave imaging (HR-MMWI) has the potential to provide affordable noninvasive tissue diagnostic information due to its ample depth of penetration and significant differentiation (41). An HR-MMWI system was utilized to evaluate its application in *in vivo* skin cancer diagnosis. The assessment involved measuring benign and malignant skin lesions from 71 patients with various conditions such as melanoma, basal cell carcinoma, squamous cell carcinoma, actinic keratosis, melanocytic nevi, angiokeratoma, dermatofibroma, solar lentigo, and seborrheic keratosis (41).

The downscaling of sensors and antenna interfaces can be performed, which stems from the short wavelength and hyperspectral characteristics of millimeter waves, making them ideal for the development of handheld or point-of-care devices. As a result, a handheld configuration can be designed instead of a benchtop HR-MMWI system, offering *in vivo* imaging of skin tissue at a remarkably low production cost. This will help dermatologists and dermatologic surgeons acquire images of cancer tissues that resemble histopathological analysis. This can be performed either before a biopsy or after tumor excision, enabling the prompt identification and removal of any remaining tumor tissue.

This portable, real-time imaging tool allows dermatologists to efficiently see more patients in multiple rooms. By utilizing MMWI, the reduction in unnecessary biopsies can exceed 50%. HR-MMWI requires less training and expertise from users. This rapid classification of lesions within 20 s represents a groundbreaking clinical advancement in the detection and management of skin cancer.

**Figure 12:**
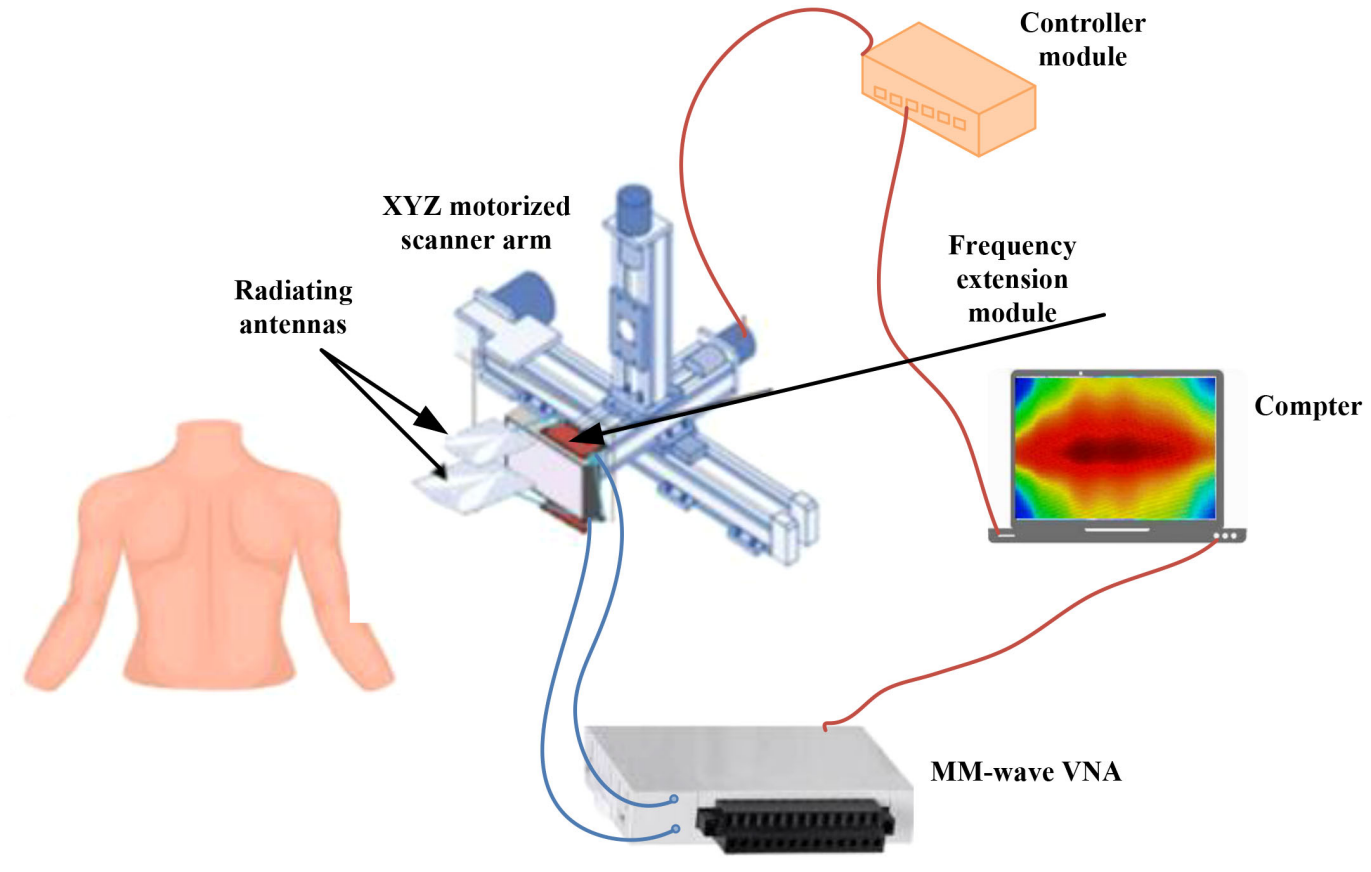
A framework for of an ultra-wideband millimeter-wave imaging. With this system, a synthetic bandwidth of 98 GHz can be achieved. To transmit and record backscattered signals, two sub-band antennas are positioned in front of the target at each scanning step (41). Reproduced from (41), licensed under CC BY 4.0.

**Figure 12** depicts the newly developed ultra-wideband millimeter-wave imaging system, which operates within a bandwidth of 98 GHz (12–110 GHz) (41). During each scanning step, two sub-band antennas are positioned in front of the target to transmit signals within their respective sub-band frequency ranges and capture the backscattered signals.

The workflow involves image reconstruction, processing, depth estimation, 3D PCA-based feature extraction, feature classification, and cancer detection based on malignancy scores. Initially, a multivariate statistical analysis based on PCA is employed to automatically identify the most significant features.

PCA condenses nearly 1,000 intensity variables from raw tissue images into six principal components (PCs), each representing a linear combination of the original variables. The number of PCs is determined from the millimeter-wave images using singular value decomposition (SVD) (41). Once the PCs are chosen, the scores and loadings are aggregated across all points and all PCs.

The PC scores were collected and used as input for the classifier model. Various linear and nonlinear classifiers, including linear discriminant analysis (LDA), K-nearest neighbors (KNN) with different K-values, linear support vector machine (LSVM), Gaussian SVM (GSVM) with different margin factors, and multilayer perceptron (MLP), were tested using all possible combinations of features (41).

The classifiers were selected based on the dataset size to ensure high reliability and robustness in classifying lesion types.

The reported results showed that optimal classification performance was obtained using five PCA components with an MLP model, achieving 97% sensitivity and 98% specificity. These findings demonstrate that real-time millimeter-wave imaging can effectively differentiate malignant from benign skin lesions, offering diagnostic accuracy comparable to standard clinical assessments and existing imaging techniques.

### 7.3 Real-time detection of brain cancer during surgical operations using spatio-spectral classification of hyperspectral images

Treating brain cancer surgically poses a substantial challenge in neurosurgery (42). Tumors often infiltrate diffusely into the surrounding normal brain, complicating their accurate identification visually. Surgery remains the primary treatment for brain cancer, and achieving precise and complete tumor removal can enhance patient survival rates.

Hyperspectral imaging is a noncontact, non-ionizing, and non-invasive tool used during surgery to visualize brain tumors in real time, assisting neurosurgeons in tumor resection. Reference (42) presents a classification method that utilizes both spatial and spectral features within hyperspectral images. This method aids neurosurgeons in precisely identifying tumor boundaries during surgical procedures.

**Figure 13:**
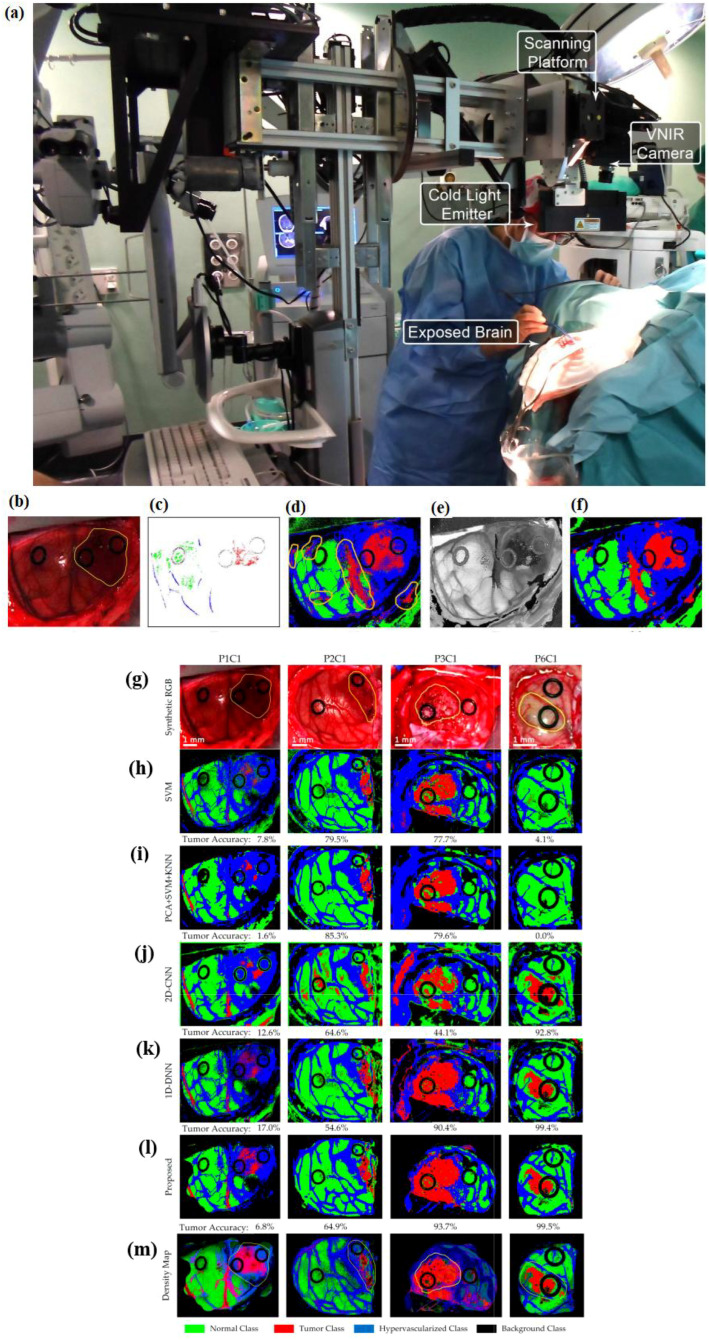
**(a)** A hyperspectral acquisition system as an intra-operative system (utilized during a neurosurgical procedure). An optimized spatial–spectral supervised classification algorithm is used, its results in each step of which are as follows. **(b)** Synthetic RGB images generated from the hyperspectral cubes. **(c)** The supervised classification training with golden standard maps. **(d)** Supervised classification maps after applying the SVM algorithm. **(e)** The hyperspectral cubes’ FR-t-SNE single-band visualization. **(f)** Classification maps optimized spatially after using the KNN filter (from (42)). Reproduced from (42), licensed under CC BY 4.0. Four test hyperspectral images’ classification maps with tumor accuracy mentioned below each map. **(g)** The highlighted area in yellow shows the synthetic RGB image with tumor **(h–l)**. The results of multiclass classification maps via using SVM, PCA + SVM + KNN, 2D convolutional neural network (2D-CNN), one-dimensional 2D deep neural network (1D-DNN), and the proposed framework, respectively (44). Reproduced from (44), licensed under CC BY 4.0. Green shows normal tissue, red shows tumor tissue, and hypervascularized tissue is shown in blue; black indicates the background. **(m)** Density maps resulting from the surgical aid visualization algorithm. The probability values (determined with the majority voting algorithm) are shown through colors (adapted from [43)]. Reproduced from (43), licensed under CC BY 4.0.

**Figure 13a** shows the intra-operative hyperspectral acquisition system. The classification framework comprises five main stages: data pre-processing, dimensionality reduction, spatial–spectral supervised classification, unsupervised clustering segmentation, and hybrid classification (42). **Figures 13b–f** present the outcomes of each phase in the optimized spatial–spectral supervised classification of the patients (42).

**Figure 13b** shows synthetic RGB images generated from the hyperspectral cubes, and **Figure 13c** depicts the reference standard maps employed for supervised classification training. **Figure 13e** presents the FR-t-SNE representation of the hyperspectral cube using a single band (42). These images highlight distinct areas on the brain surface, making it easier to identify their borders, including the tumor area.

The FR-t-SNE outcomes, combined with probability scores from the supervised classification maps, are inputted into the KNN filter. After the KNN filtering process, the spatially optimized classification map is obtained, as shown in **Figure 13f**. The regions belonging to each class in the images have been made more uniform, resulting in coherent classification maps. While the distinction between the supervised classification maps and the spatially optimized classification maps may be subtle to the naked eye (**Figures 13d,f**), this homogenization is an important process that enhances the final phase of the cancer detection algorithm (42).

During the spatial–spectral supervised classification stage, a comparison was made between the sequential time results from a CPU implementation and the time results achieved using a hardware accelerator (42). The accelerated version of the algorithm requires transmission time due to the connection between the computer and the hardware accelerator. When the hardware accelerator is used in the spatial–spectral supervised classification stage, it results in an average speed-up factor of 26.83×. During surgery with the utilized system in (42), it takes approximately 1 min to produce a classification map of the captured scene. Some false-positives have been absorbed in the results; additionally, validation of the system in clinical settings is necessary, as it has shown some misclassifications among different types of tissues, and the entire algorithm needs to be accelerated.

### 7.4 Real-time enhanced brain tumor delineation using an intraoperative visualization system with hyperspectral imaging

Hyperspectral imaging allows for the capture of a broad spectrum of wavelengths across the electromagnetic spectrum from the surfaces of scenes observed by sensors. By leveraging this data along with a range of classification algorithms, it is possible to accurately identify specific materials or substances present in each pixel. Reference (43), during neurosurgical operations (to distinguish tumor tissue from brain tissue), exploits the characteristics of hyperspectral imaging to develop a prototype which can enhance the precise outlining of tumor boundaries and improve the results of surgery.

For real-time processing, during surgical procedures, a hardware accelerator is utilized in conjunction with a control unit (43). This hardware accelerator expedites the hyperspectral brain cancer detection algorithm. A labeled dataset is used as the training data for the supervised phase, demonstrating that the system can distinguish between normal and tumor tissue in the brain during *in vivo* analysis. The system provides results within approximately 1 min during surgery, presenting a practical tool that could enhance excision and potentially enhance patient outcomes. The acquisition system incorporates a software platform that has been created by integrating three distinct software development kits (7). These kits are from two types of hyperspectral cameras and a stepper motor controller (7). The VNIR initiates the capture process from right to left across the platform equipped with the stepper motor. After capturing with VNIR, the stepper motor halts at the final position to stabilize the system for a few milliseconds before setting speed. Subsequently, NIR begins capturing from left to right on the platform. The stepper motor repositions the scanning platform to the central position. This technique accelerates the acquisition process by three times compared with the original software. The control unit manages preprocessing, HKM clustering, and the majority voting algorithm. Simultaneously, the hardware accelerator oversees the spatial–spectral supervised classification stage, incorporating PCA and SVM algorithms.

Due to high computational demands for the processing of thematic maps (which are from a validation database of hyperspectral images), it is carried out by the hardware accelerator (7). Both the unsupervised and supervised stages run concurrently, and then the control unit runs the majority voting algorithm to produce the final HELICoiD three maximum density (TMD) map (43). The brain tumor is marked red in the TMD map. This creates an RGB representation based on the top three major probabilities per cluster derived from the HKM clustering algorithm. This image is shown to the neurosurgeon through the hyperspectral processing interface.

### 7.5 Real-time deep-learning-based framework for *in vivo* identification of glioblastoma tumor using hyperspectral images of human brain

To ensure accurate guidance for tumor removal in real time during neurosurgery, it is essential to develop a method that does not rely on labeling. Hyperspectral imaging can aid surgeons without the need for any contrast agent. Reference (43) proposes a framework to process *in vivo* human brain tissue with hyperspectral images which is based on deep neural networks. A human image database is used for the evaluation of the framework. This framework produces a thematic map that outlines the brain’s parenchymal area and pinpoints the tumor’s location. A pipeline is used for data processing. This pipeline provides a density map in which gradient colors are used to represent normal, tumor, and hypervascularized tissues (43). The results obtained using the proposed framework in **Figure 13g** are promising, particularly for patient 6, where identifying the tumor location with the naked eye was extremely challenging.

A synthetic RGB image is shown in **Figure 13g**. In this image, yellow lines are utilized to outline the tumor area. **Figures 13h–l** display the results of the multiclass classification maps using SVM, PCA + SVM + KNN, 2D convolutional neural network (2D-CNN), one-dimensional 2D deep neural network (1D-DNN), and the proposed framework in (43), respectively. Normal tissue, tumor tissue, and hypervascularized tissue are represented by green, red, and blue colors, respectively, while black indicates the background. **Figure 13m** showcases the density maps generated by the surgical aid visualization algorithm, which are derived from the optimal threshold set for the tumor class.

The colors in these density maps have been modified according to the probability values obtained from the majority voting algorithm. The deep learning pipeline achieved an overall multiclass classification accuracy of 80%, surpassing the performance of traditional SVM-based methods. The proposed framework (43) employs a DNN for classification due to its shorter execution time. The CNN takes approximately 1 min to process each hyperspectral cube, significantly longer than the DNN, which completes the task in approximately 10 s per cube. The training dataset had significantly fewer samples in the tumor class compared with the other classes, which led to an equal number of samples across all classes. This balance effectively minimized the overall training duration (43). However, the use of real-time image fusion has some limitations, such as the substantial expenses associated with these systems, along with the extra time needed from physicians for examinations and image fusion.

### 7.6 Hyperspectral imaging for esophageal cancer

Studies have further demonstrated the value of hyperspectral and spectral imaging for esophageal cancer, a disease where conventional white-light endoscopy often fails to detect flat or early-stage lesions (44–46). They provide concrete, disease-specific evidence supporting the clinical value of AI-enhanced optical imaging.

Reference (44) proposed a spectrum-aided visual enhancement (SAVE) framework combining hyperspectral imaging (HSI) with deep-learning-based object detection using YOLO architectures to improve early esophageal cancer detection. The study processed more than 2,000 white-light endoscopic images, generating SAVE images via calibrated spectral reconstruction and training six YOLO variants (v3–v8, Scaled YOLOv4, YOLOv6–7). Quantitative analysis demonstrated that SAVE images significantly improved model performance compared with standard white-light images. Using the SAVE technique, YOLOv8 improved dysplasia detection accuracy to 82.1% compared with 80.9% for standard WLI. Dysplasia recall increased from 70.4% to 79.5%, and the F1-score rose from 75.2% to 80.8%, indicating stronger lesion detection capability. For SCC, SAVE raised recall to 81.3% and improved the F1-score from 83.7% to 87.0%. Moreover, YOLOv8 achieved mAP50 values of 84.1% for dysplasia and 86.7% for SCC, compared with 68.3% and 76.5% using WLI, confirming substantial performance gains with spectral enhancement, The authors highlighted the feasibility of incorporating SAVE + YOLO as a computer-aided detection (CAD) module in clinical endoscopic workflows to aid early lesion localization and reduce operator dependency. Future improvements should focus on expanding dataset diversity, standardizing spectral calibration, and conducting multi-center real-time validation to ensure robustness across imaging conditions and populations.

Reference (45) evaluated the effectiveness of a SAVE to augment white-light endoscopic images for the early detection of esophageal cancer. They trained five deep-learning object detection models (YOLOv9, YOLOv10, YOLO-NAS, RT-DETR, and Roboflow 3.0) on a dataset of images (normal, dysplasia, and SCC) and found that SAVE consistently outperformed conventional WLI. Notably, F1 scores for SCC detection rose from 84.3% to 90.4% with YOLOv9, and dysplasia precision increased from 72.4% to 76.5%. These results suggest that SAVE + YOLO frameworks hold promise for integration into clinical endoscopic workflows, enabling more accurate real‐time lesion detection and improved patient outcomes. For full translation, further work is needed on external validation, real‐time latency, and performance under varied imaging conditions.

Reference (46) investigated an AI-assisted “precision imaging” pipeline combining the hyperspectral conversion of standard white light and narrow-band endoscopic images with YOLOv5 detection using images from patients with normal esophagus, dysplasia, and squamous cell carcinoma. The HSI-enhanced models (both HSI-WLI and HSI-NBI) showed marked improvements: for instance, accuracy rose from ~78% (RGB-WLI) to ~86% (HSI-WLI), and the sensitivity rates for SCC and dysplasia also improved into the mid-80s. These results suggest that hyperspectral conversion + YOLOv5 holds substantial promise to aid in early esophageal cancer detection, particularly in clinics with varying imaging equipment. To move toward clinical workflow usage, further efforts in latency reduction, robustness to variable imaging conditions, and large-scale prospective validation are necessary.

## 8 Radiomics and deep learning in multimodal imaging

In this section, the first deep-learning-based PET and MRI image fusion for cancer diagnosis is discussed. Then, radiomics-based cancer diagnostics as well as image-omic fusion-based cancer diagnosis are described.

### 8.1 PET/MRI fusion for breast cancer

For advanced breast cancer to determine patient responses to neoadjuvant chemotherapy (NAC), CNNs with PET and MRI images as input have been used (47). CNNs are employed for data classification, and AlexNet, a CNN variant, optimizes computational efficiency and boosts accuracy through the integration of dual convolution layers, enabling a comprehensive evaluation of the response to NAC. To determine the presence or absence of a complete chemotherapy response, it has been shown that CNNs improve this. The dataset used in the study was limited in size. CNNs, while capable of analyzing complex image features, typically necessitate a larger dataset to achieve optimal performance. Therefore, to overcome this limitation, employing K-fold validation is important. The dataset exhibited a significant imbalance between responders and non-responders, potentially leading to an overestimation of accuracy. Such an imbalance may yield results that are highly misleading. Additionally, unlike the conventional method, the CNN method needs a larger sample population since it did not incorporate changes between the baseline and interim images. In this study of 56 advanced breast cancer patients, a deep learning PET/MRI model achieved high predictive performance for neoadjuvant chemotherapy response, with AUCs of 0.886 and 0.980, surpassing conventional imaging metrics. The best traditional parameter, ΔSUV (change in standardized uptake value, reflecting metabolic activity reduction), reached an AUC of 0.805, with up to 83% sensitivity and 80% specificity, highlighting its value for early and accurate treatment response prediction.

### 8.2 AI-powered radiomics in CT and MRI

Radiomics is a powerful tool for converting digital medical images such as CT and MRI into mineable, high-throughput data to improve clinical decision-making. While radiomics provides a non-invasive method of analyzing the entire tumor volume, in conventional biopsies, due to sampling limitations, it is common not to fully capture tumor heterogeneity. By quantifying gray-level intensities and spatial pixel distributions, radiomics enables the assessment of tumor heterogeneity; it has shown potential in predicting histological grade preoperatively. Texture analysis, a key component of radiomics, provides a quantitative approach to interpreting tissue heterogeneity and improves diagnostic capabilities, particularly in pancreatic lesions (48). CT texture analysis is an applicable tool in tumor identification, staging, and monitoring treatment response. In pancreatic ductal adenocarcinoma (PDAC), early detection is a critical need, as most cases are diagnosed at advanced stages, resulting in a low survival rate (48, 49). To distinct early-stage from late-stage PDAC, the use of radiomics is introduced (48). It uses contrast-enhanced CT scans from 71 patients with histologically confirmed PDAC. Analysis Kit software is used for radiomics feature extraction. Key predictive features are selected via the Mann–Whitney *U* test, univariate logistic regression, and the minimum redundancy maximum relevance (mRMR) algorithm. A radiomics model is constructed via a random forest classifier. Moreover, 10-fold leave-group-out cross-validation (LGOCV) is used for model validation. A radiomics model was developed using a random forest algorithm based on the nine most informative radiomic features. The model achieved strong discriminative performance, yielding an accuracy of 97.7%, sensitivity of 97.6%, specificity of 97.8%, positive predictive value of 98.4%, and negative predictive value of 96.8%. The findings show that the CT-based radiomics model is a promising, non-invasive method to differentiate between early- and late-stage PDAC. For full clinical utility, large-scale, multi-center prospective studies are required for further validation.

In (50), to provide personalized treatment, a method for preoperative risk classification in patients with endometrial endometrioid adenocarcinoma has been developed. To this end, an MRI-based radiomics model that combines traditional radiomics features with deep learning-derived features was introduced. The model showed good diagnostic performance, including on an external validation set, supporting its clinical functionality in risk stratification before surgery. Addressing risk classification in this specific cancer subtype and including external validation are key strengths of the study. However, there are some challenges, such as it being a retrospective single-center study prone to selection bias and missing clinical data, which may have affected model accuracy despite the use of interpolation techniques. Radiomic features (such as intensity, morphology, texture, and wavelet patterns) capture tumor phenotype and microenvironment characteristics that are complementary to clinical, treatment-related, and genetic data (50). The study analyzed data from 168 patients, dividing them into a training cohort of 95 cases and a validation cohort of 73 cases. The radiomics nomogram demonstrated strong predictive performance, with an area under the curve (AUC) of 0.923 in the training cohort and 0.842 in the validation cohort. Overall, the nomogram outperformed both the conventional radiomics model and the deep-learning-based radiomics approach. As a result, combining radiomic features with clinical and pathological variables can improve prognostic model performance, particularly in complex cancers where imaging heterogeneity presents ongoing challenges. To provide robust, generalizable models, prospective, multi-center studies with large cohorts are required.

### 8.3 AI-based image-omics integration

For effective treatment, accurate diagnosis of cancer subtypes is essential, as each subtype has distinct pathological features and therapeutic responses. While deep learning has achieved significant utility in computer vision, its application—for instance, for lung cancer subtype diagnosis—is limited. This is because between slide images of various subtypes, there are only subtle differences. Moreover, deep models tend to overfit when there is high-dimensional genomic data with limited sample sizes. Therefore, integration of image and genomic data is a challenging task (51).

To address these challenges, for improved lung cancer subtype classification (52), introduces LungDIG, a hybrid deep network. This network integrates histopathological images with genomic data. Using a fine-tuned Inception-V3, LungDIG segments whole-slide images into patches and extracts patch-level feature models. It uses a feature combination strategy (that maintains diversity across subtypes) to reduce false-positives from non-diagnostic regions. To derive genomic features from copy number variation data, an attention-based nonlinear extractor is used. Then, an attention-based multilayer perceptron (MLP) is used for image and genomic feature fusion for final classification. Experiments on TCGA lung cancer datasets demonstrate that LungDIG provides both high diagnostic accuracy and high interpretability. For accurate cancer diagnosis and prognosis prediction, it is important to provide complementary information. To this end, pathological images and molecular omics data are used (53). Efficient integration of these heterogeneous modalities can provide deeper insights into the complex mechanisms of cancer. However, in many multimodal fusion methods—due to differences in representation learning—the strength of each modality varies across tasks, leading to suboptimal performance. To address this, a multimodal balanced fusion method designed for both diagnosis and prognosis prediction (named MBFusion) is introduced (53). To extract features from molecular omics data, MBFusion employs two tailored graph convolutional networks. A ResNet-based architecture is also used to extract features from pathological images. Attention mechanisms are used to enhance the image features, and clustering is applied to retain deep, informative representations and ensure that both modalities are balanced in their capabilities. Then, a cross-attention Transformer is used for feature integration. After that, the fused representation is used in a multi-task learning setup for cancer subtype classification and survival analysis. MBFusion provides up to a 10.1% performance improvement across three evaluation metrics as well as offers strong interpretability, highlighting its potential clinical application.

### 8.4 Cancer study through molecular-level insights with single-cell transcriptomic analysis

Beyond imaging-based AI applications, recent computational advances have extended to the transcriptomic dimension of cancer analysis. Reference (54) developed a reference-free framework to identify transcriptomic events in cancer cells using single-cell RNA sequencing (scRNA-seq) data, providing an effective alternative to conventional alignment-based pipelines. Their approach overcomes a key limitation in cancer genomics—the inability of traditional methods to detect unannotated transcripts, splicing variants, and repeat-derived RNAs that contribute to tumor heterogeneity. Using esophageal carcinoma and glioblastoma datasets, the authors applied an alignment-free workflow combining k-mer decomposition, Limma Voom differential analysis, and annotation. Functional enrichment and survival modeling revealed many novel contigs linked to oncogenic pathways. They further predicted tumor-specific neoantigens from previously unmapped sequences, expanding the immunogenic landscape of cancer. This study demonstrates that alignment-free, AI-assisted transcriptomic analysis can uncover complex molecular features—such as novel splicing events, intronic activity, and long non-coding RNAs—that often escape detection in standard analyses. The approach complements imaging-based diagnostics by providing molecular-level insights into tumor progression and immune microenvironment interactions. In the long term, combining reference-free transcriptomic inference with AI-driven imaging offers a unified, multi-omics perspective to understand cancer biology and advance precision oncology.

## 9 Discussion

In this paper, we aim to review and summarize the latest literature on real-time AI-based cancer diagnosis. The review is centered on different imaging methods as well as image fusion techniques. We discuss the pros and cons of the approaches reported in the literature. We summarize these challenges as follows. Moreover, **Table 2** summarizes all the discussed real-time methods, their clinical workflow, technical parameters, maturity level, and quantitative performance.

**Table 2:** Summary of real-time imaging and AI-based diagnostic modalities in oncology.

Modality/technique	Clinical application	AI integration/algorithm	Quantitative performance	Key advantages	Limitations	Clinical status/maturity	Clinical workflow/remarks
Ultrasound fusion imaging (TRUS/MRI, CT/US)	Real-time navigation and lesion localization (prostate, liver)	Automatic registration and landmark detection	TRUS/MRI: detected cancer in 26/52 MRI-suspected cases; +61% positive rate vs. systematic biopsy. HCC <3 cm detection improved from 78.8% → 90.5% with real-time US + CT/MR fusion	Real-time, radiation-free, enhances targeting precision	Tissue deformation, registration error, operator dependency	Early clinical use	Enables image-guided biopsy and intra-operative fusion with MRI/CT
Mass spectrometry pen	Intraoperative tissue classification	LASSO + ANN	Accuracy 92%–97%; analysis <10 s	Label-free; rapid intra-op feedback	Requires contact; sterilization limits	Pilot clinical	Real-time metabolic profiling of tissue
Diffuse reflectance spectroscopy (DRS)	GI and esophageal cancer tissue classification	SVM/random forest	Accuracy 93.9% (gastric); 96.2% (esophageal)	Low-cost, portable; label-free	Limited penetration depth	Translational	Integrates with endoscopes for optical biopsy
Optical coherence tomography (OCT/PR-OCT)	Colorectal and esophageal optical biopsy	RetinaNet with ResNet-18 backbone	Sensitivity 100%; specificity 99.7%; AUC 0.998 (*n* ≈ 26k)	Near-histologic resolution	Requires GPU; small FOV	Early clinical validation	Enables high-speed optical biopsy
AI polyp detection (Endo-CRC)	Colorectal invasion depth classification	Three-tier CNN (ResNet-50 + custom CNN3)	Accuracy 91.61%–93.78% (internal), 88.65% (external); FPS 45; video accuracy 100%	Real-time; expert-level diagnostic performance	GPU-dependent; needs multi-center validation	Early clinical	Intra-procedure CADx decision support
Shear-wave elastography (SWE), RTE	Prostate cancer detection	Elasticity-based quantitative analysis	Detection 69.5% vs. 80.5% (systematic biopsy); relative sensitivity 0.92; positive cores 21% vs. 11%, combined detection 45.5% vs. 39.5%; risk ratio 1.18	Quantitative stiffness mapping; noninvasive, Improved detection with combined approaches	Operator-dependent; limited to accessible organs, Improved detection with combined approaches	Clinical use	Guides targeted biopsy using stiffness map
Neuromorphic AI (CM1K Chip)	Cervical cancer cytology	Hardware-accelerated kNN (Neuromem CM1K)	Preprocessing time: 335.5 ms (normal single-cell), 136.3 ms (abnormal); training 18 μs per neuron	Ultra-low latency; energy-efficient	Limited features; basic preprocessing	Preclinical	Real-time cytology classification chip
Fluorescence imaging (GAINS)	Tumor resection and sentinel node mapping	Real-time NIR fluorescence overlay	Sensitivity 100% in sentinel lymph node detection	Real-time intraoperative navigation	Requires dye; hardware calibration	Clinical pilot	AR-guided fluorescence surgery
Fluorescence lifetime endoscopy (FLE)	Breast and skin cancer delineation	Random forest classifier on FLIm parameters	Sensitivity 89%; specificity 93%; overall accuracy 97.5% (≥75% training)	Label-free; high spatial precision	Not yet validated in humans	Preclinical	Real-time tumor margin classification
Fluorescence confocal microscopy (FCM)	Digital pathology (prostate biopsy)	AI-assisted feature extraction	Concordance accuracy 95.1% with H&E	Instant stain-free pathology	Small field of view; shallow depth	Pilot clinical	Enables remote pathology review
Hyperspectral imaging (HSI) — Weng et al.	Early esophageal SCC/dysplasia	YOLOv5/YOLOv8	Accuracy 82.1; recall 79.5%; F1 = 80.8% for dysplasia	High diagnostic accuracy; spectral–spatial fusion	Computational load; limited dataset	Pilot	Real-time endoscopic lesion highlighting
Spectral imaging (SAVE)—Chang et al.	Early esophageal SCC/dysplasia	YOLOv9/YOLOv10/YOLO-NAS/RT-DETR	Sensitivity 88%; precision 90%; F1 90.4%	Works on standard endoscopes	Small sample size	Early validation	Adds AI overlay to WLI endoscopy
Precision imaging—Yang et al.	Esophageal cancer (WLI & NBI)	YOLOv5 on HSI-converted data	Accuracy 86%; mAP 0.83; sensitivity SCC 88%; dysplasia 86%	Compatible with existing endoscopes	Limited validation	Translational	Adapts HSI analysis to NBI/WLI modes
PET/MRI radiomics	Breast cancer NAC response	AlexNet-based CNN	AUC baseline 0.886 → interim 0.980; ΔSUV AUC 0.805 (sensitivity 83%, specificity 80%)	Quantitative predictive insight	Retrospective dataset	Clinical validation	Predicts therapy response pre-op
CT radiomics (PDAC)	Early vs. late PDAC classification	Random forest	Accuracy 97.7%; sensitivity 97.6%; specificity 97.8%; PPV 98.4%; NPV 96.8%	Non-invasive; high accuracy	Retrospective	Translational	Risk stratification and treatment planning
MRI radiomics (endometrial)	Pre-op risk classification	Deep radiomics model	AUC 0.923 (train); 0.842 (validation)	Non-invasive biomarker	Prospective validation needed	Early clinical	Assists surgical decision support

### 9.1 Real-time ultrasound image fusion

-There are still challenges to address using augmented reality clinically in surgeries involving soft tissue organs since surgical targets and surrounding anatomies can move or deform during surgery due to respiration, heartbeat, or surgical manipulation. AI models trained on intraoperative videos and images can learn typical deformation patterns and adjust AR overlays accordingly.
-Robot-assisted targeting provides enhanced accuracy. However, incorporating 3D real-time digital registration and targeting is crucial for supporting focal therapy. AI-based landmark detection can be incorporated into modern fusion software to further improve registration accuracy and reduce operator variability.
-Although liver US is an essential tool in clinical medicine, it has limitations when it comes to diagnosing or performing interventional procedures on focal liver lesions. Contrast-enhanced ultrasound (CEUS) significantly improves the visualization and characterization of liver lesions by enhancing vascular contrast in real time. CEUS has excellent diagnostic accuracy in differentiating focal liver lesions.

### 9.2 Different real-time spectroscopy and optical-imaging-based cancer diagnosis

- In a multicenter setting, real-time optical assessment cannot eliminate the possibility that features would have been scored differently by other endoscopists. The training session where definitions were discussed may have reduced significant interobserver variation. Additionally, variation in rater assessment could be beneficial for generalizing the results to real-world practice.
-The real-time setting led to some LNPCPs being biopsied before optical assessment. It is important to consider the evaluation of individual endoscopists’ accuracy, as the number of LNPCPs assessed per endoscopist was too small to provide individual data. This limitation also prevented the exploration of whether factors such as the type of scope used influenced accuracy.
-The number of T1 colorectal cancers was still limited, so the score chart was restricted to discriminating T1 colorectal cancers from non-invasive polyps without considering different submucosal invasion depths. Future studies should evaluate this by possibly updating the risk score with other features. The limited number of T1 colorectal cancers unavoidably led to less precise estimates.
-One major limitation of the current PR-OCT research is that all imaging procedures were performed *ex vivo*, which does not capture the complexity of the *in vivo* human environment. Additionally, the system was evaluated on a limited number of abnormalities and did not include hyperplastic polyps, which are common but rarely resected due to their benign nature. Differentiating adenomatous from hyperplastic polyps in future *in vivo* studies would have significant clinical value. The system also lacked sufficient testing on radiation- or chemotherapy-treated tumors, which may require expanded classification categories. Finally, the small training dataset may reduce the system’s ability to accurately predict abnormal lesions.
-Future efforts will include both hardware and software integration of PR-OCT into the endoscope, fine-tuning the network, and evaluation in the *in vivo* setting.
-The CADx tool cannot identify sessile serrated polyps, a recently recognized polyp type with likely neoplastic potential. Another limitation is the learning curve of the colonoscopists during the study period, as the prospective study design may contribute to the underestimation of the CADx performance.
-Future cost-effective studies in colonoscopy with CADx may investigate whether the extended procedure time is justified by the potential benefits of reduced polypectomies.
-To advance the Raman technique for early cancer detection, collaborative efforts are needed among scientific teams comprising biophysicists, clinicians, and biomedical engineers. Further validation of Raman spectroscopy’s clinical utility is essential. Additionally, the adoption of new technologies as they emerge is crucial for enabling real-time, *in vivo* diagnosis across various types of cancer.
-Removing noise and artifacts from real-time Raman spectra of live tissues remains a challenge, as they can interfere with interpretation in pathology. Novel designs of Raman fiber optic probes and data handling algorithms are necessary to enable a fully automated spectral analysis.
-Future investigations should focus on addressing the challenge of fusing images with large anatomical variation due to organ deformation before the widespread use of fusion imaging in managing gastrointestinal malignancies. Non-rigid registration algorithms can be useful to solve this issue.
-Conducting a multicenter trial would facilitate the recruitment of participants from a broader population. However, the method for correlating tumor location on the specimen was constrained by the histopathology protocols. Standardized pathology mapping and digital tagging techniques can improve tumor localization across centers, overcoming limitations from variable histopathology protocols.
-H&E slides had to be used in conjunction with images of the sliced tissue and the entire specimen to manually label tumor areas. This approach may have resulted in mislabeling some data points on the border between the tumor and healthy tissue. To minimize mislabeling at tumor borders, future studies should use co-registered digital pathology and automated AI-based segmentation to align histology slides with whole-specimen images, improving labeling accuracy at the tumor–normal interface.

### 9.3 Real-time elastography-based cancer diagnosis

-In order to prove that RTE-targeted biopsy is superior to systematic biopsy, there are inadequate reasons. However, combining both techniques enhances the prostate cancer detection accuracy. Future studies should focus on validating this combined approach through large-scale, prospective trials.
-SWE may miss or underestimate cancer size and Gleason score, contributing to a high false-positive rate. Biopsy, as the reference standard, lacks precise location correlation with SWE, causing stiff areas on SWE to sometimes not match actual cancer, leading to misdiagnoses. Developing improved SWE algorithms to differentiate cancerous stiffness from benign tissue and integrating multi-parametric data to reduce false-positives are needed.
Additionally, there is a need to address the lack of data on inter- and intra-observer variability, as this can impact the accuracy of prostate SWE imaging. Conducting standardized training and implementing automated or AI-assisted analysis to reduce inter- and intra-observer variability in prostate SWE imaging are required. To improve accuracy, operators should release pressure on the transducer when a stiff area is detected and image the lesion from different angles to avoid misdiagnosis.
-Moving forward, the focus should be on objective comparisons between systematic and RTE-targeted biopsy approaches to detect clinically significant prostate cancer. Employing deep learning systems for real-time analysis and diagnosis under complex conditions is crucial for clinical AI applications. Multimodal data processing has been shown to enhance the robustness of AI-aided diagnosis.

### 9.4 Real-time different fluorescence-image-based cancer diagnosis

-Further improvements are needed to enable the detection of microscopic lesions in the surgical field, which might otherwise be missed, and to possibly prevent damage to nearby uninvolved vital structures such as nerves.
To ensure the success of FLE based on PH as a practical medical device, two key issues need to be addressed: *in vivo* pH measurement and intravenous administration for fluorescein. These issues should be discussed and resolved by comparing *in vivo* pH measurement between the proposed system and a commercial pH measurement with intraoperative intravenous fluorescence injection in further studies.

### 9.5 Real-time hyperspectral imaging and cancer diagnosis

-The HR-MMWI system’s functionality can be improved by integrating all imaging antennas and their corresponding circuits into a single framework using microwave integrated circuit technology.
-Utilizing hardware acceleration platforms such as GPUs or FPGAs to implement the full brain cancer detection algorithm is possible. This implementation should explore the design space to achieve the optimal balance between real-time execution, memory usage, and power dissipation using heterogeneous platforms.

### 9.6 Real-time PET/MRI image deep learning breast cancer

-Further studies are necessary to enhance the model’s ability to inform clinical treatment decisions.
-Future study is needed to determine how the risk score chart can be improved to be ultimately used for clinical decision-making. Through this clinical decision making, the type of endoscopic resection and whether to proceed to surgery instead of endoscopy must be clarified.
-The network must be trained on a larger number of patients. Assessing the novel “wait and watch” rectal cancer treatment management strategy could be a potential future improvement. This approach enables treatment responders with no residual cancer to be safely monitored through imaging instead of surgery, thereby preserving their quality of life.

### 9.7 AI-driven radiomics and image-genomics fusion

-While not real time in execution, radiomics and image–genomics fusion frameworks extend the value of AI beyond intraoperative use. These tools allow for comprehensive tumor characterization, risk classification, and prognostic modeling based on preoperative imaging and molecular profiles. Their integration into clinical workflows could enhance surgical planning and stratification decisions in real-time settings.

### 9.8 General limitations and barriers to adoption of different technology and imaging

Several barriers still hinder the clinical adoption of AI-based real-time imaging. Algorithmic challenges include overfitting, data imbalance, and limited domain generalizability, which collectively reduce model robustness and reproducibility. Overfitting happens when deep learning models are trained on small or single-center datasets, leading to excellent training accuracy but poor generalization to new populations or imaging paradigm. Strategies such as dropout regularization, ensemble learning, and transfer learning from large-scale repositories have been applied to mitigate this effect. Data imbalance is another fundamental obstacle, as clinical datasets typically contain a predominance of normal or benign samples compared with malignant lesions. This imbalance biases model predictions toward the majority class and limits sensitivity for rare pathologies. To address this, techniques such as data augmentation, class-weighted loss functions, and synthetic image generation using generative adversarial networks (GANs) or diffusion models have been explored, although the lack of large, balanced, multi-center datasets continues to restrict scalability. Domain adaptation—the process of aligning model performance across different imaging devices, acquisition protocols, and institutions—is recognized as critical for real-world deployment. Variations in scanner hardware, image resolution, and population demographics often decrease model accuracy when applied outside the training set. Emerging methods such as adversarial domain adaptation, self-supervised pretraining, and federated learning are making models learn device- and site-invariant representations, consequently improving cross-institutional reliability.

Beyond these algorithmic challenges, workflow and integration barriers are also important. Some AI-assisted systems prolong procedures—for instance, computer-aided diagnosis modules can add up to 40 s per lesion assessment during colonoscopy—leading challenges in fast-paced clinical settings. Interoperability issues also arise from proprietary vendor platforms and the lack of standardized data formats for multimodal image fusion, complicating integration into existing hospital infrastructures. Moreover, the scarcity of well-annotated, publicly accessible multimodal datasets remains a major limitation. Most current studies are single center and often involving fewer than 100 patients, with inconsistent annotation protocols across institutions. Without multi-center validation and standardized data, progress in AI-based real-time imaging will remain confined to proof-of-concept systems rather than scalable clinical deployment. Overcoming these constraints—through balanced datasets, robust domain adaptation, and smooth workflow integration—will be critical to transitioning AI-assisted imaging from experimental innovation to reliable clinical use.

## 10 Conclusion

This review has explored the convergence of real-time imaging, artificial intelligence, and image fusion technologies in advancing cancer diagnosis and intraoperative guidance. Across various clinical domains, technologies such as computer-aided navigation systems—particularly those integrated with robotic platforms and organ-tracking capabilities—are improving the precision and outcomes of minimally invasive surgeries. Incorporating real-time imaging modalities such as ultrasound into fusion systems further facilitates multimodal comparisons and often reduces radiation exposure for both patients and clinicians.

Optical diagnostic methods are rapidly evolving, with tools like diffuse reflectance spectroscopy (DRS), Raman spectroscopy, and fluorescence-based imaging demonstrating promise in real-time tissue classification and tumor margin assessment—for instance, DRS has successfully distinguished malignant from healthy tissue in gastrointestinal cancers through *ex vivo* studies, paving the way for *in vivo* applications pending further validation. Similarly, Raman spectroscopy, when paired with sensitive imaging techniques like fluorescence endoscopy, offers a powerful avenue for early detection and intraoperative decision-making, though its full clinical translation requires robust multicenter trials.

Shear wave elastography (SWE) provides complementary information by assessing tissue stiffness, correlating well with tumor aggressiveness as reflected by Gleason scores. While systematic biopsy remains standard, real-time elastography shows improved core-specific detection rates, suggesting value in combined approaches. Technologies such as fluorescence confocal microscopy (FCM) are also emerging as stain-free, real-time diagnostic tools with potential applicability across multiple cancers due to their digital output and remote reporting capabilities.

Although radiomics and multi-omics fusion approaches are not inherently real time, they provide essential preoperative insights that can inform intraoperative decisions. Their integration into AI-enhanced diagnostic pipelines exemplifies a holistic approach to cancer management, merging computational precision with personalized care.

Despite these promising advances, practical challenges persist. Tissue deformation during surgery, variability in patient positioning, and landmark registration errors can impact the accuracy of real-time image fusion. Machine learning, particularly AI-based landmark detection and convolutional neural networks, offers a path forward by improving registration reliability and supporting diagnostic precision, as demonstrated in breast cancer response evaluation using PET/CT and MRI. To fully realize the potential of these innovations, future research must focus on expanding image databases, optimizing algorithms, validating findings in large-scale clinical settings, and developing hardware accelerators to support real-time processing.

Moving forward, a structured roadmap is needed to guide the clinical translation of AI-based real-time and multimodal imaging. The first priority is providing a large, multi-center prospective trials that validate AI performance across diverse populations and imaging platforms—such as ultrasound fusion, hyperspectral endoscopy, and fluorescence guidance—to confirm robustness and reproducibility. In addition, the standardization of data acquisition and annotation protocols is important, which will ensure interoperability and facilitate cross-institutional algorithm benchmarking. The development of open, benchmark datasets for real-time optical, ultrasound, and fusion modalities are needed to enable fair model comparison and transparent performance reporting. Clinically, smooth workflow integration and regulatory alignment—including explainable AI interfaces, latency optimization, and human-in-the-loop quality control—will be required to building clinician trust and ensuring safe deployment. Finally, collaboration among clinicians, engineers, data scientists, and regulatory partners will be vital to providing these translational milestones and transforming AI-based imaging from experimental tools into a dependable component of precision oncology and intraoperative decision-making.

In summary, for these approaches to achieve routine clinical use, several steps remain critical, namely: (1) development of robust algorithms that overcome data imbalance and improve explainability, (2) creation and sharing of large, standardized, multi-modal datasets with multi-center validation, (3) optimization of workflows to ensure that AI systems integrate smoothly into surgical and diagnostic routines without adding significant time, and (4) deployment of hardware accelerators such as GPUs and FPGAs to enable real-time performance. Addressing these unmet needs through coordinated research and infrastructure development will allow AI-based imaging systems to evolve from experimental tools into practical clinical solutions that directly benefit patients.
